# Broad and Efficient Control of *Klebsiella* Pathogens by Peptidoglycan-Degrading and Pore-Forming Bacteriocins Klebicins

**DOI:** 10.1038/s41598-019-51969-1

**Published:** 2019-10-28

**Authors:** Erna Denkovskienė, Šarūnas Paškevičius, Audrius Misiūnas, Benita Stočkūnaitė, Urtė Starkevič, Astra Vitkauskienė, Simone Hahn-Löbmann, Steve Schulz, Anatoli Giritch, Yuri Gleba, Aušra Ražanskienė

**Affiliations:** 1 Nomads UAB, Geležinio vilko 29A, LT-01112 Vilnius, Lithuania; 2 Vilnius University, Institute of Biotechnology, Saulėtekio al. 7, LT-10257 Vilnius, Lithuania; 3 Lithuanian University of Health Sciences, Department of Laboratory Medicine, Eivenių g. 2, LT-50161 Kaunas, Lithuania; 4 Nomad Bioscience GmbH, Biozentrum Halle, Weinbergweg 22, D-06120 Halle (Saale), Germany

**Keywords:** Plant biotechnology, Bacterial toxins, Recombinant protein therapy, Clinical microbiology

## Abstract

Gram-negative bacteria belonging to the genus *Klebsiella* are important nosocomial pathogens, readily acquiring resistance to all known antibiotics. Bacteriocins, non-antibiotic antibacterial proteins, have been earlier proposed as potential therapeutic agents for control of other Gram-negative species such as *Escherichia*, *Pseudomonas* and *Salmonella*. This study is the first report describing pore-forming and peptidoglycan-degrading bacteriocins klebicins from *Klebsiella*. We have identified, cloned, expressed in plants and characterized nine pore-forming and peptidoglycan-degrading bacteriocins from different *Klebsiella* species. We demonstrate that klebicins can be used for broad and efficient control of 101 of the 107 clinical isolates representing five *Klebsiella* species, including multi-drug resistant pathovars and pathovars resistant to carbapenem antibiotics.

## Introduction

Klebsiellae are nonmotile, rod-shaped, Gram-negative bacteria, encased by a polysaccharide capsule providing resistance against many host defense mechanisms^1^. Three species in the genus *Klebsiella* are commonly associated with illness in humans: *K*. *pneumoniae*, *K*. *oxytoca*, and *K*. *granulomatis*. Recently it was discovered that two more *Klebsiella* species, *K*. *variicola* and *K*. *quasipneumoniae*, also can cause deadly infections^2^.

*Klebsiella* is an important pathogen in nosocomial infections and is responsible for many clinical syndromes including pneumonia, bacteremia, thrombophlebitis, urinary tract infection, cholecystitis, diarrhea, upper respiratory tract infection, wound infection, osteomyelitis, and meningitis^3^. The use of invasive devices, use of respiratory support equipment, of urinary catheters, and treatment by antibiotics are risk factors causing potential infection^4^. The principal pathogenic reservoirs for infection are the gastrointestinal tract of patients and the hands of hospital personnel^1^.

*Klebsiella pneumoniae* is one of the six ESKAPE pathogens (*Enterococcus faecium*, *Staphylococcus aureus*, *Klebsiella pneumoniae*, *Acinetobacter baumannii*, *Pseudomonas aeruginosa*, *Enterobacter* spp.) causing hospital infections, which readily developed resistance to antibiotics^5^. In 2016 an outbreak of nosocomial pneumonia was reported in China, where five surgical patients died from infection with carbapenem-resistant *K*. *pneumoniae* (CRKP) ST_11_ strains that had acquired a virulence plasmid. Such new strains are simultaneously hypervirulent, multidrug resistant, and transmissible and could pose a serious threat to public health^6^.

In light of the ever-increasing drug resistance shown by many pathogens, the development of a new generation of antimicrobial substances is urgently needed, in particular to control Gram-negative bacteria. An alternative approach to antimicrobial treatment is to use the well-characterized species-specific colicin-like bacteriocins which are produced by a wide range of Gram-negative bacteria^7^. The potential use of such bacteriocins as narrow spectrum antimicrobials, in particular *E*. *coli* colicins and *P*. *aeruginosa* pyocins, have been explored in several studies^8–13^.

Colicin-like bacteriocins, first described in *E*. *coli*, kill only closely related bacteria, belonging to the same species or genera. Their mechanisms of action are diverse, including pore-formation, DNAse or RNAse activity, or disruption of peptidoglycan biosynthesis in the periplasm. The narrow target range of colicins has been shown to be due to the presence of specific receptors at the surface of the sensitive strains to which colicin binds before killing. The three steps of colicin action have been described: the colicin molecule causes killing after binding to a specific receptor on the outer membrane and being translocated through the cell envelope by either the Tol or TonB machinery to its target. All colicins are organized into three domains in relation with the three steps of their action: reception, translocation, and killing. Binding to specific receptors located in the outer membrane, translocation across the cell envelope, and cytotoxic activities are dependent on the central, N-terminal, and C-terminal domains, respectively^14^.

So far, the *Klebsiella* colicin-like bacteriocins, klebicins, have received very little attention and only few studies have been published, all describing klebicins with presumed nuclease activity^15–17^. Several putative ColM-type bacteriocins have been recently identified *in silico* in *Klebsiella* genomes^18^. We describe here several new pore-forming and peptidoglycan-degrading bacteriocins from different *Klebsiella* species and demonstrate that they can be used for broad and efficient control of *Klebsiella* pathogens, in particular multi-drug resistant pathovars.

## Results

### Identification and analysis of klebicin sequences

**Figure 1:**
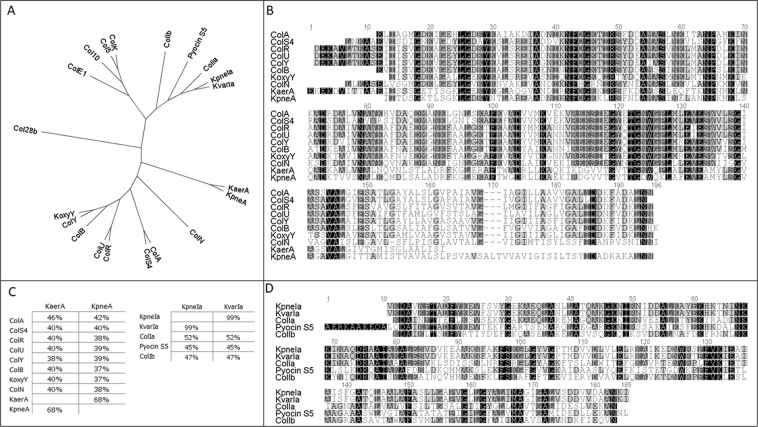
Sequence analysis of pore forming domain-containing colicins and their *Klebsiella* homologues. (**A**) Phylogenetic tree of predicted pore-forming domains (pfam01024; Geneious tree builder) of pore-forming colicins, pyocin S5 and klebicins. KpneA (SAV78255.1), KaerA (WP_063414841.1), KoxyY (WP_024273778), KvarIa (KDL88409), KpneIa (BAS34675), Col28B (CAA44310.1), ColE1 (AAA87379.1), Col10 (CAA57998.1), Col5 (CAA61102.1), ColK (Q47502.1), ColIb (AAA23188.1), Pyocin S5 (WP_003115311), ColIa (WP_001283344.1), Col N (P08083.1), ColA (P04480.1), ColS4 (CAB46008.1), ColR (AGV40809.1), ColU (CAA72509.1), ColB (P05819.3), ColY (AAF82683.1). (**B**) Clustal W amino acid sequence alignment of pore-forming domains of colicin A-like proteins. (**C**) Amino acid identity between pore-forming colicins and klebicins (Pfam14859). (**D**) Clustal W amino acid sequence alignment of pore-forming domains of colicin Ia-like proteins.

Putative *Klebsiella* bacteriocin amino acid sequences were retrieved from NCBI by BLAST search using as queries ColM (pfam 14859) and pore forming domains of ColA, ColIa and ColU (pfam01024). After the analysis of BLAST results we have selected the five most divergent pore-forming domain containing proteins from various *Klebsiella* species (*K*. *pneumoniae* SAV78255.1, *K*. *aerogenes* WP_063414841.1, *K*. *oxytoca* WP_024273778, *K*. *variicola* KDL88409, *K*. *pneumoniae* BAS34675) and four colicin M-like putative bacteriocins (*K*. *pneumoniae* EWD35590.1, *Klebsiella sp*. WP_047066220, *K*. *variicola* CTQ17225.1, *K*. *aerogenes* WP_015367360.1). The predicted pore-forming domain sequences of these putative bacteriocins were subjected to Clustal W amino acid sequence alignment and Geneious tree builder analysis along with all pore-forming domains of known pore-forming colicins and pyocin S5. Two major groups of *Klebsiella* pore-forming domain-containing proteins can be distinguished: *K*. *pneumoniae* BAS34675 and *K*. *variicola* KDL88409 are most related to colicin Ia, and *K*. *aerogenes* WP_063414841, *K*. *pneumoniae* SAV78255 and *K*. *oxytoca* WP_024273778 belong to the group of proteins similar to colicin A (Fig. 1A). WP_024273778 is most similar to colicin Y with 75% of amino acid identity between their pore forming domains (Fig. 1B,C). Based on these findings, we named putative *Klebsiella* bacteriocins according to the host species name and to the most related colicin: KpneA (*K*. *pneumoniae* SAV78255.1), KaerA (*K*. *aerogenes* WP_063414841.1), KoxyY (*K*. *oxytoca* WP_024273778), KvarIa (*K*. *variicola* KDL88409) and KpneIa (*K*. *pneumoniae* BAS34675). Amino acid sequences of predicted pore-forming domain of KpneIa and KvarIa are 52% identical to ColIa (Fig. 1C,D). KpneIa and KvarIa are almost identical, but KpneIa contains a stretch of 52 a.a. in the central part of the protein, which is absent in KvarIa. KaerA and KpneA pore-forming domains have 46% and 42% identity with ColA, respectively (Fig. 1B,C).

**Figure 2:**
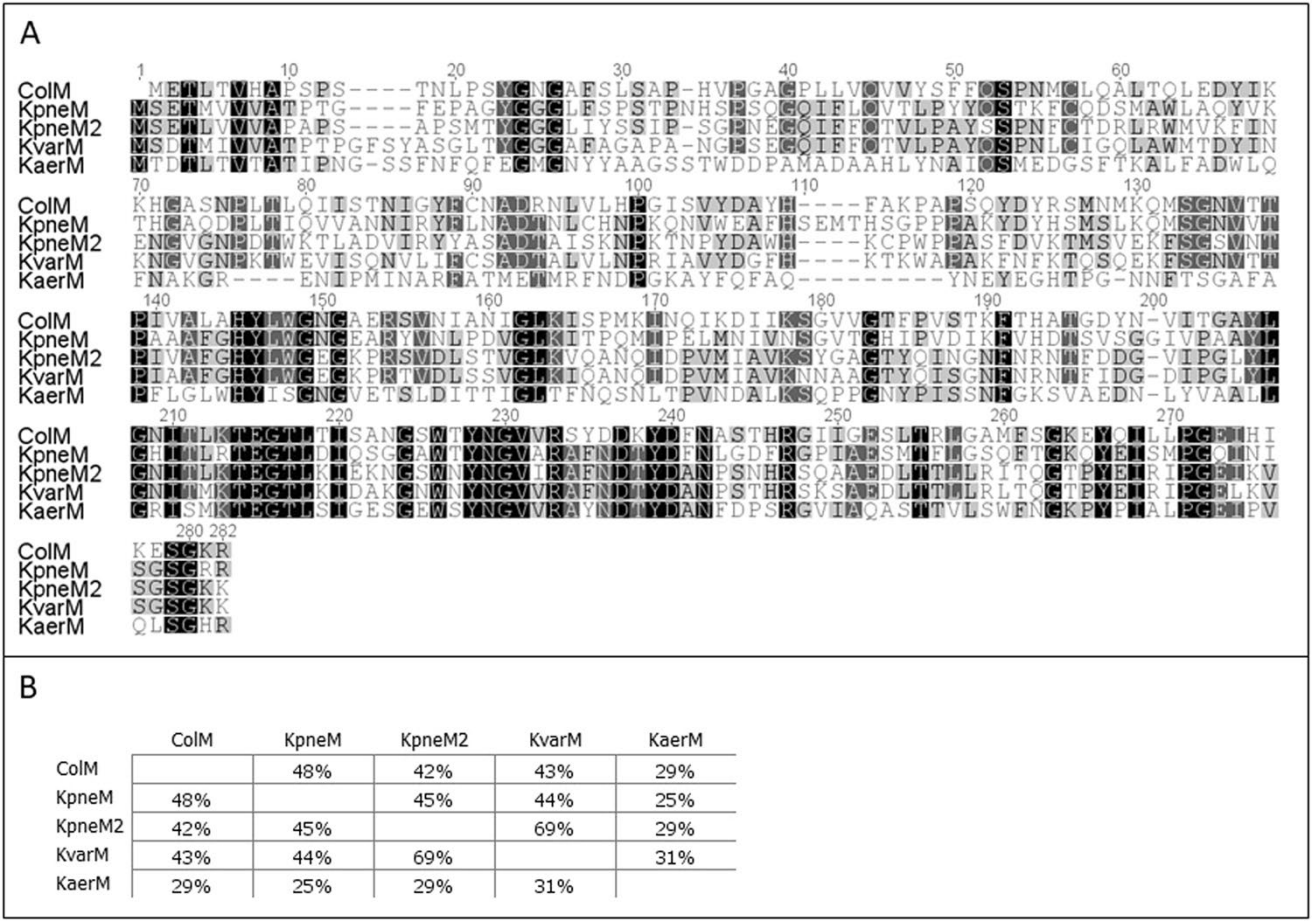
Sequence analysis of colicin M and its *Klebsiella* homologues. (**A**) Clustal W amino acid sequence alignments. ColM (AAA23589.1), KpneM (EWD35590.1), KpneM2 (WP_047066220), KvarM (CTQ17225.1), KaerM (WP_015367360.1). (**B**) Amino acid identity between colicin M and klebicins.

The four colicin-M-like *Klebsiella* proteins show different degrees of homology with colicin M over full length of the protein in range of 29 to 48 percent of identity, the highest sequence similarity noticed in the carboxy-terminal domains (Fig. 2). All four proteins were also named according to the host species name: KpneM (*K*. *pneumoniae* EWD35590.1), KpneM2 (*Klebsiella sp*. WP_047066220), KvarM (*K*. *variicola* CTQ17225.1) and KaerM (*K*. *aerogenes* WP_015367360.1).

### Klebicins are expressed in and recovered from green plants with high yield

**Figure 3:**
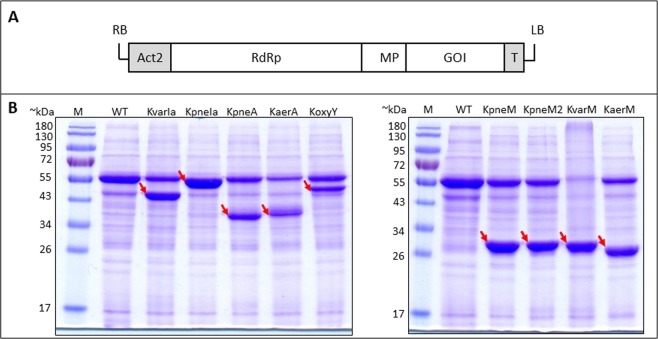
Klebicin expression in plants. (**A**) Schematic presentation of T-DNA region with klebicin expression cassette. RB – right T-DNA border, Act2 – *A*. *thaliana* actin promoter, RdRp – RNA-dependent RNA polymerase, MP – tobacco mosaic virus movement protein, GOI – gene of interest, T – *A*. *tumefaciens* nopaline synthase terminator, LB – left T-DNA border. (**B**) Expression of klebicins in *N*. *benthamiana* leaves. Plant material (50 mg, pooled samples of three leaves) was harvested at 4 (KaerA) or 5 (all other klebicins) days post infiltration (dpi), ground in liquid nitrogen, extracted with 50 mM Tris-HCl, 300 mM NaCl 15 mM CH_3_COONa, 3 mM DTT (pH 7.5), and denatured at 98 °C for 10 min. Solutions containing 5 µg of protein were resolved in 12% polyacrylamide gel for Coomassie staining. M – PageRuler Prestained protein ladder (Thermo Fisher Scientific), WT – crude extract of non-infiltrated *N*. *benthamiana* leaves, KvarIa, KpneIa, KpneA, KaerA, KoxyY, KpneM, KpnM2, KvarA, KaerM – extracts of *N*. *benthamiana* leaves, infiltrated with klebicin expression constructs. Bands corresponding to recombinant klebicins are marked by arrows.

All nine synthetic klebicin genes with plant-optimized codons, containing no added tags, were cloned into the magnICON® tobacco mosaic virus-based vector pICH29912 (Fig. 3A)^19^. The resultant binary expression vectors were used to transform *A*. *tumefaciens*. Klebicins were transiently expressed in plants by infiltrating transformed agrobacteria strains into leaves of young *N*. *benthamiana* plants by syringe infiltration. The SDS-PAGE and Coomassie staining analysis of the extracts of soluble proteins of infiltrated plant leaves revealed that all nine klebicins are efficiently expressed in plants and are detected in the gel as very intense supplementary bands (Fig. 3B). The weights of polypeptides observed in electrophoresis approximately correspond to the expected theoretical molecular weights (KvarIa – 43.4 kDa, KpneIa – 48.5 kDa, KpneA – 40 kDa, Kaer A – 39 kDa, KoxyY – 48.7 kDa, KpneM – 30.3 kDa, KpneM2 – 29.7 kDa, KvarM – 29.8 kDa and KaerM – 29 kDa) (Fig. 3B). The expression levels of individual klebicins varied in a range of 2,7-4,4 mg/g FW, the highest expression levels were achieved for the two *K*. *pneumoniae* M-type klebicins KpneM2 and KpneM (Table 1).

**Table 1:** Purification method, obtained yields and purity of plant-expressed klebicins.

Klebicin	Purification method	Klebicin amount in crude extract (μg/g FW)	Yield of purified protein (μg/g FW)	Purity %
KpneM	Phenyl > DS > Q	3239 ± 224	1134 ± 54	99.8 ± 0.3
KpneM2	Phenyl > DS > Q	4448 ± 347	920 ± 55	99.5 ± 0.5
KvarM	Phenyl > DS > Q	2423 ± 146	535 ± 35	98.1 ± 1.0
KpneA	Phenyl > DS > SP	2677 ± 163	337 ± 26	97.2 ± 1.2
KaerA	SP > DS > Q	2718 ± 227	468 ± 46	96.3 ± 0.7
KvarIa	Phenyl > DS > SP	2697 ± 149	629 ± 31	98.9 ± 1.0

### Plant-expressed klebicins exhibit broad antimicrobial activity against different *Klebsiella* species

**Figure 4:**
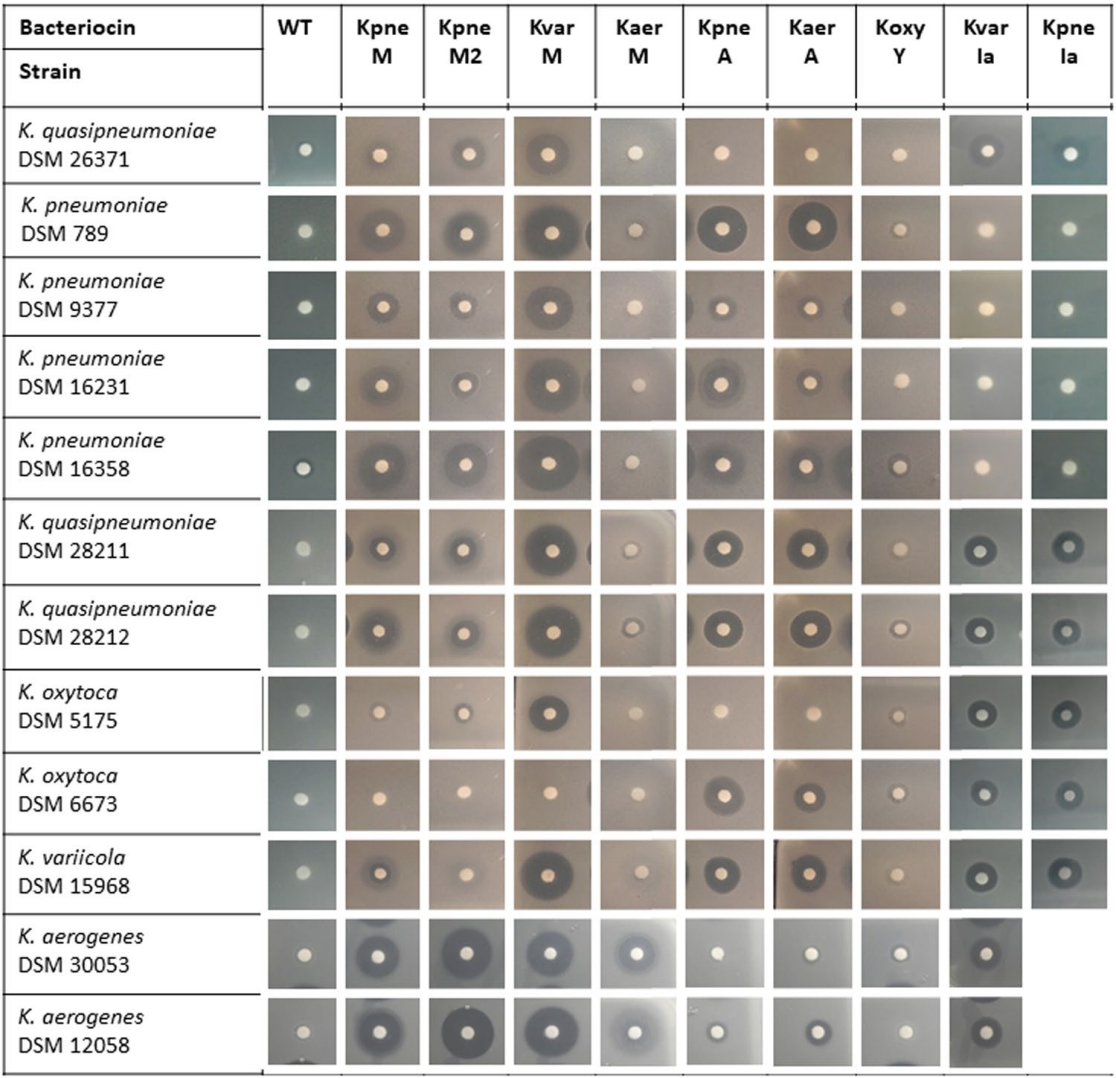
Evaluation of klebicin activity against *Klebsiella* strains in soft-agar overlay assay. Aliquots of 20 µl (~50 µg) of protein crude extracts were spotted on 6 mm Whatman paper discs on *Klebsiella* lawn and incubated overnight.

As the next step, we tested the activity of the crude bacteriocin-containing plant extracts in soft-agar overlay assay with twelve *Klebsiella* strains belonging to different species (*K*. *pneumoniae*, *K*. *quasipneumoniae*, *K*. *oxytoca*, *K*. *variicola* and *K*. *aerogenes*). Two of the tested bacteriocins, KoxyY and KaerM demonstrated perceptible but very narrow inhibition zones on the lawn of several tested strains, with KaerM forming larger hazy inhibition zones only on *K*. *aerogenes* lawn. Because of the weak and/or narrow activity, these two proteins were not included in subsequent experiments (Fig. 4).

All seven remaining bacteriocins formed large inhibition zones on the lawn of several tested *Klebsiella* species and strains. All twelve tested strains were inhibited by several bacteriocins. The three ColM-like proteins demonstrated broadest activity spectrum and similar activity pattern, targeting eleven out of twelve tested strains. However, KvarM formed significantly larger inhibition zones than both *K*. *pneumoniae* ColM-like bacteriocins (KpneM and KpneM2). The two ColA-like proteins KpneA and KaerA also demonstrated a very similar activity pattern, although the zone diameter was different for some of the tested strains. And finally, both ColIa-like proteins KvarIa and KpneIa demonstrated an identical activity pattern (Fig. 4). All bacteriocins formed inhibition zones on the strains belonging to all five different *Klebsiella* species with exception of KvarIa and KpneIa. The two ColIa-like proteins had no effect on neither of four tested *K*. *pneumoniae* strains.

Thus, we confirmed that all cloned colicin-like proteins are indeed active *Klebsiella* colicin orthologues – klebicins. This experiment with crude plant extracts allowed us to only semi-quantitatively compare activities of different klebicins because of differences in expression levels. However, it permitted us to choose the apparently most efficient bacteriocins for purification and further experiments: KpneA, KaerA, KvarIa, KpneM, KpneM2 and KvarM. KpneIa was not included in further experiments since it displays an identical spectrum as KvarIa.

### Purification of klebicins

KpneA, KaerA, KvarIa, KpneM, KpneM2 and KvarM bacteriocins were purified to homogeneity by protein chromatography (up to 99.8% protein purity, Table 1). Quite pure KpneM, KpneM2 and KvarM proteins were obtained after single-step hydrophobic interaction chromatography (HIC), but for best results a second purification step by anion exchange chromatography was included. KpneA and KvarIa were also purified by HIC as a first step, but then followed by cation exchange chromatography. KaerA was purified by two steps of ion exchange chromatography, cation exchange column as a first step and anion exchange column as second step (Suppl. Text S1, Suppl. Figs S1, S2 and Table 1).

All purified klebicins contain only 0.2-3.7% of protein impurities, as determined by capillary gel electrophoresis. The yields of individual klebicins after purification are in range of 0.34-1.1 mg/g of fresh weight. The purification of klebicins with greatest expression levels resulted in the biggest final yields and also best quality of purified proteins (highest purity) (Table 1).

We checked for the loss of the activity of purified klebicins by determining the minimal inhibitory concentrations (MIC) for two *K*. *pneumoniae* and *K*. *variicola strains*, tested with crude extracts in agar diffusion assays (Fig. 4). It appears that purified klebicins are highly active: for susceptible strains, MICs of the klebicins were in range of 0.1-0.8 µg/ml (Suppl. Table S1).

### Klebicins as antimicrobial non-antibiotic alternative for control of MDR Klebsiella

As a next step, all six purified klebicins were tested against a larger panel of clinical *Klebsiella* isolates: 89 *K*. *pneumoniae* and 11 *K*. *oxytoca* strains, in total one hundred clinical *Klebsiella* isolates from the Lithuanian Health Science University hospital in Kaunas. All isolates in the panel were resistant to at least one antibiotic and 68% of isolates were multidrug resistant (resistant to three or more antimicrobial classes), but all were sensitive to carbapenems (Suppl. Table S2).

**Figure 5:**
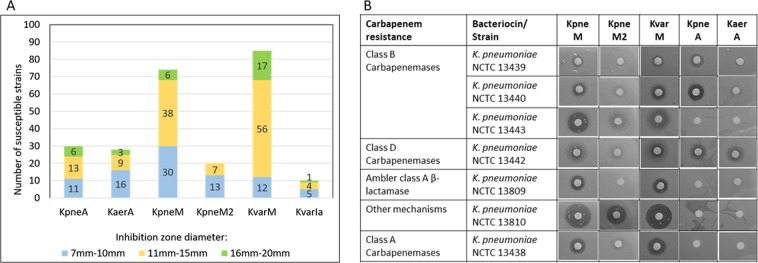
Sensitivity of clinical isolates to plant-expressed klebicins. (**A**) 100 clinical *Klebsiella* isolates (89 *K*. *pneumoniae* and 11 *K*. *oxytoca* strains) were tested in soft agar overlay assay applying 20 µg of each klebicin. The strains susceptible to each klebicin are grouped by the size of the inhibition zone. (**B**) Seven NCTC antimicrobial resistance reference *K*. *pneumoniae* strains with different mechanisms of carbapenem resistance were tested in soft agar overlay assay applying 20 µg of each klebicin.

92% in total and 88% of MDR isolates were sensitive to at least one klebicin. KvarM demonstrated the broadest spectrum of activity, since 85% of strains were sensitive to this klebicin (Fig. 5A and Suppl. Table S2). KpneM was not far behind, targeting 74% of tested strains, in general with slightly smaller inhibition zones. The specificity of KvarM and KpneM activity spectra were largely overlapping, but KvarM targeted 11 strains more that KpneM, and only one strain resistant to KvarM was sensitive to KpneM (Fig. 5A and Suppl. Table S2). In contrast, the third M-type klebicin KpneM2 was much less active, targeting only 20% of strains. Both ColA-like klebicins KpneA and KaerA targeted 30% and 28% of strains, respectively, with partially overlapping profiles. 9 strains resistant to KaerA were sensitive to KpneA, and 7 strains resistant to KpneA were sensitive to KaerA. KpneA also in general formed larger inhibition zones. KvarIa had the narrowest spectrum of activity and targeted only 10% of all strains, 6 strains of *K*. *oxytoca* and 4 strains of *K*. *pneumoniae* (Fig. 5A and Suppl. Table S2).

As the tested panel did not include carbapenem resistant strains, a separate study was done with NCTC antimicrobial resistance reference *K*. *pneumoniae* strains with different carbapenem resistance mechanisms (Fig. 5B). Despite the small strain panel in this test, the obtained data mirrors the results obtained with the panel of carbapenem-sensitive strains. KvarM and KpneM are active against all seven tested strains, KpneM2 forms distinct inhibition zone on one strain and small hazy zones on several strains. KpneA targets three strains, and KaerA forms distinct zones on one strain.

### Klebicins exhibit high antimicrobial activity on planktonic and biofilm cells of *Klebsiella*

We next performed a more detailed analysis of klebicin activity in liquid medium and on young, one day-old biofilms with five representatives of different *Klebsiella* species: *K*. *pneumoniae*, *K*. *quasipneumoniae*, *K*. *oxytoca*, *K*. *variicola* and *K*. *aerogenes*.

**Figure 6:**
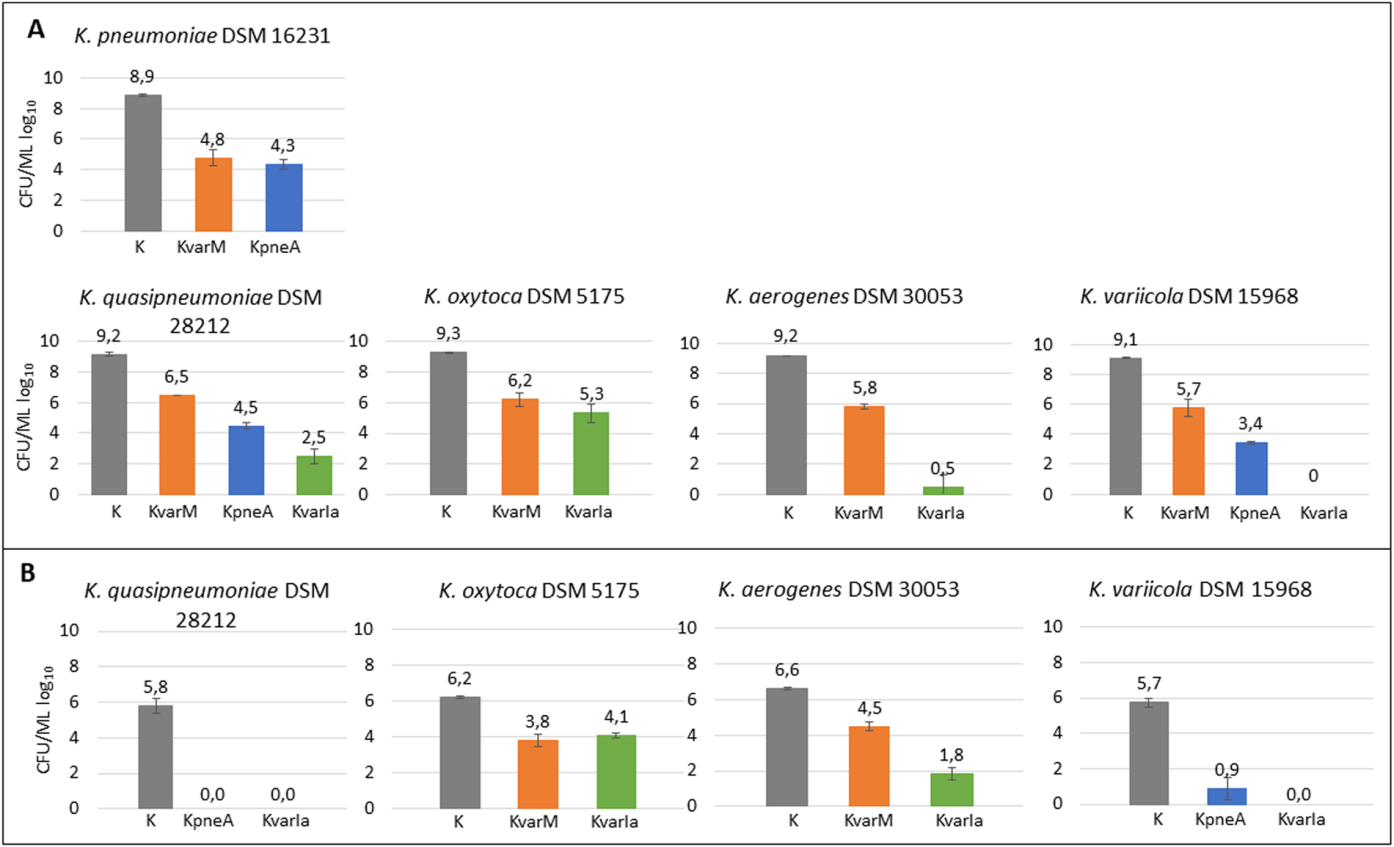
Evaluation of klebicin activity against *Klebsiella* in liquid cultures (**A**) and on biofilms (**B**). (**A**) *Klebsiella* overnight cultures were diluted to OD_600_ = 0.3 in CAA medium, then klebicins were added to bacterial cultures at 5 µg/ml and bacteria were further incubated for 5 h at 30 °C or 37 °C with shaking (200 rpm). (**B**) One day-old *K*. *quasipneumoniae*, *K*. *oxytoca*, *K*. *variicola*, *K*. *aerogenes* biofilms grown in CAA medium were treated with 5 µg/ml of either of klebicins (KvarM, KpneA, KvarIa represented by orange, blue and green bars, respectively) or buffer as control (K, shown in grey bars). At the end of experiments, serial dilutions were made, plated on LB agar plates, incubated at 28 °C or 37 °C for 24 h and CFU were enumerated. Data are the mean ± SD of three independent experiments.

One best performing klebicin of each group (ColM-like, ColA-like and ColIa-like) at a concentration of 5 µg/ml was used in these assays. In liquid medium assay, klebicin KvarM inhibited the growth of all five strains, reducing the CFU number in quite similar extent – by four orders of magnitude for *K*. *pneumoniae* DSM 16231 and about three orders of magnitude for all remaining *Klebsiellae*. KvarIa inhibited four strains and was the most efficient of all three klebicins, reducing CFU by four to nine orders of magnitude, depending on the strain (as mentioned above, *K*. *pneumoniae* DSM16231 is insensitive to this klebicin). KpneA reduced CFU of three strains by 4.6 to 5.7 logs (Fig. 6A).

For biofilm assays, we used the same *Klebsiella* strains as in liquid culture assays. At first, we tested the ability of these five strains to form biofilms. Four of the tested strains formed biofilms in tested conditions, with exception of *K*. *pneumoniae* DSM 16231, thus this strain was not used for further experiments. The biofilms of all remaining four stains were treated for 20 h each with two klebicins, which showed the best results in liquid culture assays. The results obtained with biofilms quite closely reflected the results obtained in liquid culture assays, with exception of *K*. *quasipneumoniae*, for which biofilms were completely eradicated by KpneA and KvarIa. For all remaining strains, klebicin treatment efficiently decreased the numbers of CFU in biofilms to a similar extent as in liquid cultures (Fig. 6B).

### Klebicins can rescue *Galleria melonella* from lethal *Klebsiella* infection

For the initial demonstration of klebicin activity *in vivo*, we have chosen to perform *Klebsiella* challenge assay in a non-mammal animal model, *Galleria mellonella* larvae. We selected KvarIa for this assay as one of most active klebicins and *K*. *quasipneumoniae* DSM 28212 as a KvarIa-sensitive challenge strain. At first, we determined the minimal lethal dose (MLD) of the bacterial challenge strain, sufficient to kill all the larvae within 68 h (the duration of experiment), as 2.3 × 10^4^ CFU.

We next performed the challenge experiment with MLD and with two additional challenge doses, one inferior and one superior to MLD. 1.2 × 10^4^ CFU were not sufficient to kill all the larvae, as 15% of larvae still survived after 68 h. However, 2.3 and 3.2 × 10^4^ CFU were sufficient to kill all the larvae within 44 h. Injection of 10 μg of KvarIa 2 hours post infection completely rescued all the larvae infected by 1.2 and 2.3 × 10^4^ CFU. Larvae infected by the highest number of bacterial cells (3.2 × 10^4^ CFU) were rescued partially, with 85% of larvae surviving until the end of experiment (Suppl. Fig. S3).

### Identification of proteins exploited by klebicins for import into *Klebsiella* cells

In sharp contrast to some other bacteriocins, which are strictly species-specific (for example, pyocins), klebicin activity is not confined to the single species from which they are isolated, but rather to the genus. Having made this observation, we next inquired at the mechanisms involved in klebicin reception and translocation. *Klebsiella quasipneumoniae* DSM 28212, a strain with known genome sequence and sensitive to all the klebicins, was subjected to several rounds of transposon mutagenesis and pooled mutants were tested for their sensitivity towards different klebicins. 29 independent mutant clones were isolated, and transposon insertions were successfully mapped in 18 klebicin-resistant mutants. To confirm that klebicin sensitivity loss was indeed due to the mapped mutations, we performed complementation assays by ectopic expression of respective wild-type genes. The summarized results from sensitivity studies of klebicin mutants and complementation assays are presented in Supplementary Table S3.

Based on the obtained results, all klebicins with the exception of KvarIa are similar to group B colicins and use the TonB-dependent translocation pathway. All three M-type klebicins require ferrichrome receptor FhuA, TonB and ExbB for their reception – translocation, like their *E*. *coli* homologue colicin M^7,20^. Ferrichrome receptor in *Pseudomonas* FiuA is also the receptor of some *Pseudomonas* M-type pyocins^21^. KpneA and KaerA are also dependent on the TonB translocation pathway and they need in addition functional OmpC. So far, we could not identify any other putative receptor for these two klebicins (Table 2).

**Table 2:** Identified *Klebsiella* proteins involved in reception and translocation of klebicins.

Klebicin	Receptor	Mechanism of translocation	Presumptive cytotoxicity
KpneM	FhuA	TonB, ExbB	Peptidoglycan synthesis inhibitor
KpneM2	FhuA	TonB, ExbB	Peptidoglycan synthesis inhibitor
KvarM	FhuA	TonB, ExbB	Peptidoglycan synthesis inhibitor
KpneA	OmpC	TonB, ExbB	Pore forming
KaerA	OmpC	TonB, ExbB	Pore forming
KvarIa	OmpC	?	Pore forming

KvarIa-resistant transposon mutants were very difficult to obtain, and only some false-positive clones were isolated. Thus, we could identify only one protein participating in KvarIa reception – translocation, the outer membrane protein C (OmpC). OmpC mutants were selected by their resistance to KpneA and KaerA and it appeared that they are equally resistant to KvarIa.

## Discussion

We successfully cloned nine colicin-like bacteriocins from several *Klebsiella* species. Based on sequence alignments, four of the cloned klebicins are homologs of colicin M, showing moderate to higher levels of sequence identity (29-48%) with this protein. There was no published information about *K*. *pneumoniae* ColM-type bacteriocins when we started the study, but recently, Ghequire and co-workers^18^ have identified in *Klebsiella* genomes several ColM-like klebicins. Three ColM-like klebicins from our study are identical to klebicins from this independent study (KpneM is identical to Kpne CHS110, KpneM2 to Kpne e1602 and KvarM to Kvar 6A2).

Activity domains of the five remaining klebicins containing pore forming domains (pfam01024) demonstrate highest amino acid identities with colicin A (KpneA, KaerA), colicin Ia (KvarIa, KpneIa) and colicin Y (KoxyY).

Klebicin expression in plants is extremely efficient. Recently, expression of colicins and salmocins in plants have been reported^13,22^. The best expressed colicin (colK) amounted to 3 mg/g FW and the best expressed salmocin (SalE2) reached 1.7 mg/g FW. For klebicins, the highest expression levels were as high as 4.4 mg/g FW (KpneM2) and 3.3 mg/g FW (KpneM).

The klebicin activity assays showed highly positive antibacterial effects. It appears that KvarM has an exceptionally wide spectrum of activity as it could target *Klebsiella* strains belonging to *K*. *pneumoniae*, *K*. *quasipneumoniae*, *K*. *variicola*, *K*. *oxytoca*, and *K*. *aerogenes* species. It was also active against 85% of strains in the panel of antibiotic-resistant clinical *Klebsiella* isolates. In liquid culture assay, this klebicin could reduce the colony forming number count by as much as three to four logs and more than by two logs in biofilm assay. Pore-forming klebicins, although more narrowly active, were in general even more efficient in reducing viability of planktonic bacteria or bacteria grown in biofilms than peptidoglycan synthesis inhibitors and were able to achieve four to nine logs of CFU reduction in liquid cultures and two to almost six logs of CFU reduction in biofilms. KvarIa, which has demonstrated highest efficiency *in vitro*, was also tested *in vivo* in *G*. *mellonella* larvae challenge assays with very good outcome. *G*. *mellonella* larvae model has been previously used to study the activity of pyocins S2 and S5^12,23^. The *G*. *mellonella* model provides an excellent tool for pre-screening antimicrobial candidates and can help reduce the number of experiments with mammalian species^12^. Although it is not possible to compare directly the activity of KvarIa with activity of S2 or S5 in these different studies (different bacteria, different challenge doses and different bacteriocin doses were used), in all cases colicin-like bacteriocins efficiently protected larvae from death when used in range of ~0.07-0.9 µmol/kg of body weight. However, the applicability of KvarIa is currently handicapped by the fact that it is not broadly active against *K*. *pneumoniae*, although it works well on its close relative *K*. *quasipneumoniae*. We thought that genetic engineering of this klebicin with the aim to expanding its specificity to include *K*. *pneumoniae* would be worthwhile to undertake. With this task in mind, we have started to analyse the translocation mechanisms that klebicins employ to cross the *Klebsiella* outer membrane in order to exert their cytotoxic functions.

A universal feature of all colicins is their domain organization, and each colicin appears to have receptor binding, translocation and cytotoxic domains, a feature that is conditioned by the necessity of these bacteriocins to cross the outer membrane of Gram-negative bacteria^24^. Amino acid sequence alignments of pore-forming klebicins with their *E*. *coli* counterparts reveals that their cytotoxic domains show a significant degree of homology. However, as a rule, pore forming klebicins have lower molecular weights than colicins. Their amino-terminal portions, which should contain translocation and receptor binding domains, are much shorter than the respective domains of colicins. Full-length protein amino sequence alignment between pore-forming colicins and klebicins demonstrates little sequence similarity outside cytotoxic domains. The exception is amino-terminal sequence of KvarIa, KpneIa and KoxyY (up to 45 first a.a.) highly identical to amino-terminal sequence of some group A pore-forming colicins (Suppl. Fig. 4). Thus, we anticipated that the translocation mechanism of pore-forming klebicins might be different than their *E*. *coli* counterparts.

In order to find klebicin-resistant mutants, we used mini transposon mutagenesis as a simple and rapid technique^25^. We thus demonstrated that all three M-type klebicins KpneM, KpneM2 and KvarM are translocated by the mechanism similar to that of colicin M and that they need the FhuA receptor and TonB-related translocation pathway to enter the periplasm and exert their activity^20,26^.

Two klebicins, which we named KpneA and KaerA based on the similarity of their killing domain to colicin A, appeared also dependent on the TonB translocation pathway. This is in contrast to colicin A, which is translocated by TolA-dependent pathway^27^. Also, while colicin A binds to BtuB, we did not isolate any BtuB mutants resistant to KpneA or KaerA. However, both KpneA and KaerA need functional OmpC, an analog of OmpF, which participates also in ColA translocation^24^. We could not so far identify any other putative receptor for these two klebicins.

KvarIa is different from all remaining klebicins, as it appears to be functional in all TonB and ExbB mutants. Thus, based on our results, KvarIa does not use the TonB-dependent translocation pathway. Taking into account that all described colicins use either TonB or TolA as translocators, it would be expected that this protein is thus translocated by the Tol-dependent pathway. However, we did not isolate any single transpositional mutant of Tol-dependent pathway related genes that would be resistant to KvarIa. It certainly could be related to the limits of the method used, as KvarIa-resistant transposon mutants were very difficult to obtain and only some false-positive clones were isolated. Mutations with high fitness penalty might not have been obtained in the conditions used for selection. As a result, we could thus far identify only one protein participating in KvarIa reception – translocation, the outer membrane protein C (OmpC). OmpC mutants were selected by their resistance to KpneA and KaerA and it turned that they are equally resistant to KvarIa.

Further elucidation of klebicin receptors and translocators is important also for practical use of these klebicins. Klebicins are most promising to use for fighting antibiotic-resistant strains. Fighting carbapenem resistant strains would be most important and indeed all our tested carbapenem-resistant strains were sensitive to KvarM and KpneM. However, we had doubts that such strains will be targeted also by pore forming klebicins, as it has been shown that 97.1% of carbapenem-resistant *Klebsiella* strains do not express or express less OmpC or OmpF^28^. Thus, carbapenem-resistant *Klebsiella* could also be resistant to pore-forming klebicins, as all these klebicins require functional OmpC for their activity. However, three out of seven tested carbapenem-resistant strains were sensitive to KpneA, which uses OmpC as a receptor. These results exceeded our expectations, but testing a larger panel of carbapenem-resistant strains would be necessary to determine the real extent of their sensitivity to pore-forming klebicins.

Further broad studies on resistance mutation frequencies are necessary to assess the practical utility of bacteriocins as antibiotic alternatives. Our data seem to indicate that the way forward would be the use of bacteriocin cocktails that combine proteins with different receptor/translocation routes, as well as the use of bacteriocins specifically engineered for low resistance frequency. Domains from different colicin-like bacteriocins can be combined to customize their features, such as target specificity, the immunity profile, mode of action and stability^29^. Taking advantage of publicly available genome sequence information, one could envisage designing bacteriocins with a high probability of hitting a chosen pathogen of interest by targeting receptors occurring frequently in nature, preferably essential for viability or resulting in a major growth disadvantages could spontaneous loss-of-function occur, and combined with toxin (sub)types for which immunity is rare^30^.

Currently, we can conclude that we have a panel of six highly efficient plant-expressed klebicins, which can together target over 94% (101 of 107) of *Klebsiella* clinical isolates evaluated. Even without further engineering and improvement, these proteins could be further developed as an antimicrobial cocktail for their potential use against antibiotic-resistant *Klebsiella*.

## Methods

### Bacterial strains and cultures

*Klebsiella* strains were purchased from Leibniz Institute DSMZ-German Collection of Microorganisms and Cell Cultures and from Public Health England National Collection of Type Cultures (NCTC) and are described in Supplementary Information (Suppl. Table S4). Unless otherwise stated, *Klebsiella* strains were prepared by culturing in Lysogeny Broth (LB) medium (Roth) or Casamino Acids (CAA) (0.5% Bacto^TM^ Casamino acids, 5.2 mM K_2_HPO_4_, 1 mM MgSO_4_) medium (BD Biosciences) at 28 °C, 30 °C or 37 °C (as indicated in Suppl. Table 1) with shaking (200 rpm); overnight cultures were prepared by inoculation from frozen stocks. Clinical *Klebsiella* strains used for agar overlay assay have been isolated in Lithuanian University of Health Sciences, Kaunas clinics, and are described in Suppl. Table S2.

### Construction of klebicin expression vectors

The open reading frames encoding for KpneA (*K*. *pneumoniae* SAV78255.1), KaerA (*K*. *aerogenes* WP_063414841.1), KoxyY (*K*. *oxytoca* WP_024273778), KvarIa (*K*. *variicola* KDL88409), KpneIa (*K*. *pneumoniae* BAS34675), KpneM (*K*. *pneumoniae* EWD35590.1), KpneM2 (*Klebsiella sp*. WP_047066220), KvarM (*K*. *variicola* CTQ17225.1), and KaerM (*K*. *aerogenes* WP_015367360.1) optimized for expression in the host plant *Nicotiana benthamiana* were synthetized by Thermofisher Scientific (USA) and inserted as *Bsa*I-*Bsa*I fragments into pICH29912, an assembled TMV-based magnICON^®^ vector^19^ (Fig. 3A). Obtained plasmids were used to transform *A*. *tumefaciens* GV3101.

### Klebicin expression in plants

Klebicin expression in plants was performed as described in^12^ with some modifications. (*Nicotiana benthamiana* plants were grown in a growth chamber at 25 °C and 50% humidity, with a 16 h light (1500 lux) and 8 h dark photoperiod. Four-to-six-week-old plants were used for transfection with recombinant *A*. *tumefaciens*.

*A*. *tumefaciens* were grown overnight at 30 °C in LB medium containing 50 mg/l rifampicin and 50 mg/l kanamycin. *Agrobacterium* overnight cultures were sedimented at 3220 *g* for 5 min and resuspended in infiltration buffer (10 mM MES, 10 mM MgSO_4_, pH 5.5) at an OD_595_ of 1.5.

Four-to-six-week-old plant leaves were infiltrated into the abaxial side of the leaf using a syringe without a needle with a 1:100 dilution of *A*. *tumefaciens* strain containing the expression vector for each klebicin. Plant leaves were observed and collected at 4-7 dpi (days post infiltration).

### Purification of plant-produced klebicins

Preparation of crude extracts of plant-produced klebicins was performed as described in^12^ with some modifications. A small portion of frozen leaf tissue was ground into fine powder with mortar and pestle using liquid nitrogen. Prepared powder was mixed with cold extraction buffer at a ratio of 1 g of plant material to 5 ml of buffer. The suspension was kept on ice for 15-20 min. Cell debris was removed by centrifugation at 3220 *g* at 4 °C for 20 min., and the supernatant was filtered through membrane filters (pore sizes 5 µm and 0.22 µm). Obtained solution was taken as total soluble protein and applied for purification by two-step chromatography. Detailed protocols of purification are presented in Supplementary information (Suppl. Text 1 and Suppl. Fig. 1). The concentration of purified proteins was evaluated by Bradford assay or by comparison of band intensity with known BSA amount run on the same SDS-PAGE gel.

### Klebicin antimicrobial activity evaluation in liquid culture

Klebicin antimicrobial activity evaluation was performed as described by^12^, with some modifications. *Klebsiella* overnight cultures were diluted to OD_595_ = 0.3 in iron-deficient Casamino Acids (CAA) medium (BD Bioscience). Lyophilized purified klebicins were resuspended in CAA medium, added to diluted bacterial suspension and incubated for 5 hours at 30 °C or 37 °C with shaking (200 rpm). The antimicrobial activity of klebicins was evaluated by determining cell numbers of bacterial test culture. Serial 10-fold dilutions, 10^−1^, 10^−2^, 10^−3^, 10^−4^ and 10^−5^ were made, plated on LB agar plates. Upon incubation of plates for overnight at 30 °C or 37 °C, CFU were counted and cell numbers per ml of bacterial culture were calculated. All experiments were repeated 3 times.

### Soft-agar overlay assays

Soft-agar overlay assays were performed as described by^12^, with some modifications. *Klebsiella* overnight cultures were equalized to OD_595_ = 1.0 in LB medium and diluted 100-fold in 0.8% (w/v) top agar preheated in a 55 °C water bath. Mixed overlay components were poured on plates containing solid medium (LB containing 1.5% (w/v) agar). Sterile Whatman paper discs (6 mm diameter) were placed on the surface of the soft-agar medium containing bacterial test strains and respective amounts of klebicins (20 µl of crude extracts or 10 µg of purified klebicins) were applied to the discs. The plates were incubated for overnight at 30 °C or 37 °C and the diameter of klebicin inhibition zones was measured.

### Biofilm assays

Biofilms were grown as described by^12,31^, with some modifications. Briefly, *K*. *quasipneumoniae*, *K*. *oxytoca*, *K*. *variicola*, *K*. *aerogenes* strains were grown overnight in LB and diluted to OD_595_ = 0.1 with fresh CAA medium. For preparation of the biofilm growth plate, 10 µl of bacterial culture were transferred to the wells of a 96-well microtiter plate (Nalgene Nunc International, Rochester, N.Y.) containing 90 µl of CAA medium. Bacterial biofilms were formed by immersing the pegs of the biofilm detection lid, a modified polystyrene microtiter lid (Nunc TSP system), into the wells of the biofilm growth plate, followed by incubation at 30 °C or 37 °C for 20 h. For treatment with klebicins, peg biofilm detection lids were rinsed three times in sterile water, placed into microtiter plates containing 5 µg/ml of klebicins diluted in 100 µl CAA per well and incubated for 5 h at 30 °C or 37 °C, depending on the strain. After incubation with klebicins, peg lids were again rinsed three times in sterile water, placed into CAA in a sterile microtiter plate and centrifuged at 810 *g* for 30 min. The content of 6 identically treated wells was pooled for each sample and serial dilutions thereof were made and plated on LB plates for CFU enumeration. All experiments were repeated 3 times.

### *Galleria mellonella* larvae challenge experiments

*Galleria mellonella* challenge experiments were performed as described in^12^, with some modifications. *Klebsiella quasipneumoniae* strain DSM 28212 culture was grown for overnight in LB medium and diluted in 0.8% (w/v) NaCl in order to achieve a concentration of 1.2-3.2 × 10^6^ CFU/ml. 10 µl of *K*. *quasipneumoniae* culture and 10 µl of klebicin solution (1 µg/µl) were injected into the hemocoel of the fifth instar of *G*. *mellonella* larvae (Livefood UK) in proximity of the left and/or right prolegs. Klebicins were injected two hours post infection with *K*. *quasipneumoniae*. Injected larvae were incubated at 37 °C in 9 cm Petri dishes without food for up to 3 days. Caterpillars were considered dead when they displayed no movement in response to mechanical stimulus to the head, leading to distinct change in color from cream to dark brown/black. Twenty larvae were used per each treatment point.

### Transposon mutagenesis of *K*. *quasipneumoniae* DSM 28212

Transposon mutagenesis of *K*. *quasipneumoniae* strain DSM 28212 was performed as described in^25^. The suicide delivery of mini-transposons localized in pBAM1 plasmid was performed by triparental mating. The plasmid was mobilized from *E*. *coli* CC118λpir (pBAM1) donor cells into *K*. *quasipneumoniae* DSM 28212 cells with the assistance of the helper strain *E*. *coli* HB101 (pRK600). Obtained kanamycin-resistant clones were confirmed for the loss of the ampicillin resistance and their genomic DNA was used for the PCR amplification of the transposon adjacent regions, followed by sequencing, as described by Martínez-García and co-workers^25^.

### Complementation assays

*Klebsiella* genome regions, containing *ExbB*, *ExbBD*, *OmpC*, *FhuA*, *TonB* and *FimB* gene ORFs along with 5′ non-coding promoter regions, were PCR-amplified from *K*. *quasipneumoniae* DSM 28212 genomic DNA with help of Phusion DNA polymerase (Thermofisher Scientific Baltics) and ligated in pJET1.2 (Thermofisher Scientific Baltics). After sequencing, cloned fragments were excised with a pair of restriction endonucleases specific for each fragment, ligated in pACYC184 (NEB) and transformed into the respective *K*. *quasipneumoniae* mutants. The sequences of primers used and cloning strategy are described in Suppl. Table S5.

## Supplementary information

Supplementary information

## Data Availability

All data generated or analysed during this study are included in this published article and Supplementary Information.
