# Supplementary material for: Broad and Efficient Control of Klebsiella Pathogens by Peptidoglycan-Degrading and Pore-Forming Bacteriocins Klebicins
Source: Sci Rep. 2019 Oct 28;9:15422. doi: 10.1038/s41598-019-51969-1 (PMC6817936; doi:10.1038/s41598-019-51969-1)
Supplement: Supplementary file 1 — Supplementary information [file 41598_2019_51969_MOESM1_ESM.pdf]

BROAD AND EFFICIENT CONTROL OF *KLEBSIELLA* PATHOGENS BY PEPTIDOGLYCAN-DEGRADING AND PORE-FORMING BACTERIOCINS KLEBICINS

Erna Denkovskienė[1], Šarūnas Paškevičius[1,2], Audrius Misiūnas[1], Benita Stočkūnaitė[1], Urtė Starkevič[1], Astra Vitkauskienė[3], Simone Hahn-Löbmann[4], Steve Schulz[4], Anatoli Giritch[4], Yuri Gleba[4], Aušra Ražanskienė[1*].

[1] – Nomads UAB, Geležinio vilko 29A, LT-01112, Vilnius, Lithuania.

[2] – Vilnius University, Institute of Biotechnology, Saulėtekio al. 7, LT-10257 Vilnius, Lithuania.

[3] – Lithuanian University of Health Sciences, Department of Laboratory Medicine, Eivenių g. 2, LT-50161, Kaunas, Lithuania.

[4] – Nomad Bioscience GmbH, Biozentrum Halle, Weinbergweg 22, D-06120 Halle (Saale), Germany.

* – Corresponding author. Nomads UAB, Geležinio vilko 29A, LT-01112, Vilnius, Lithuania, Tel. 00370 679 65278, e-mail: ausra@nomadsbio.lt

Supplementary Table S1. Determination of klebicin MIC by microbroth dilution method[1] against *K. pneumoniae* and *K. variicola* strains. N.t. – not tested.

	K. pneumoniae DSM16358	*K. variicola* DSM15968
KpneA	>50	0.2
KaerA	>50	0.1
KvarIa	n.t.	0.4
KvarM	0.1	0.2
KpneM	0.8	0.4
KpneM2	0.8	N.t.

Supplementary Table S2. Purified lyophilized klebicins were resuspended in deionized water and applied as 10 µl drops (10 µg of protein) on 6 mm Whatman discs placed on LB plates with streaked *Klebsiella*. After overnight incubation inhibition zones were measured. All clinical isolates were also tested for resistance to following antibiotics: aminoglycosides (amikacin AMK and gentamicin GEN), penicilins (amoxicillin AMX, ampicillin AMP, ampicillin/sulbactam SAM, amoxicillin/clavulanate AMC), uredopenicilins (piperacilin PIP, piperacillin/tazobactam TZP), cephalosporins (cefoperazone CFP, cefotaxime CTX, cefuroxime CXM), fluoroquinolones (ciprofloxacin CIP), carbapenems (meropenem MEM, imipenem IPM), nitrofurans (nitrofurantoin NIT), sulfonamides (trimethoprim TMP). The isolates resistant to three or more antibiotic classes are defined as multiresistant (MDR).

No inhibition zone	Zone 7-10mm	Zone 11-15mm	Zone 16-20mm
-	+	+	+

No	Species	Ref.	Origin	MDR	Resistance	KpneA	KaerA	KpneM	KpneM2	KvarM	KvarIa
1	*K. pneumoniae*	16490	urine	MDR	AMC, AMP, CTX, CXM, CIP, GEN, NIT	-	-	+	+	+	-
2	*K. pneumoniae*	16794	bronchus	MDR	AMC, AMP, SAM, CTX, CXM, CIP, PIP, TZP	-	-	+	+	+	-
3	*K. pneumoniae*	16769	urine	MDR	AMC, AMP, SAM, CTX, CXM, CIP, GEN, NIT, TMP	-	-	+	+	+	-
4	*K. pneumoniae*	17065	trachea	-	AMP	-	-	+	-	+	-
5	*K. pneumoniae*	16871	urine	-	AMP	-	-	+	-	+	-
6	*K. pneumoniae*	16987	blood	MDR	AMX, AMP, SAM, CTX, CXM, CIP, GEN, PIP, TZP	-	-	+	+	+	-
7	*K. pneumoniae*	16866	urine	-	AMP	-	-	+	-	-	-
8	*K. pneumoniae*	16864	urine	MDR	AMX, AMP, SAM, CTX, CXM, CIP, GEN, NIT, TZP, TMP	+	+	+	+	+	-
9	*K. pneumoniae*	16806	urine	MDR	AMX, AMP, SAM, CTX, CXM, CIP, GEN, NIT, TZP, TMP	-	-	-	-	+	-
10	*K. pneumoniae*	16774	gall	MDR	AMP, SAM, CIP, PIP	-	+	+	-	+	-
11	*K. pneumoniae*	16895	pleura	MDR	AMX, AMP, SAM, CXM, PIP, TZP	-	-	+	-	+	-
12	*K. pneumoniae*	17064	bronchus	-	AMP	+	-	+	-	+	-
13	*K. pneumoniae*	16874	urine	MDR	AMP, CIP, NIT, TMP	-	-	+	+	+	-
14	*K. pneumoniae*	17173	urine	MDR	AMP, CIP, GEN, TMP	-	+	+	-	+	-
15	*K. pneumoniae*	17236	urine	MDR	AMX, AMP, SAM, CTX, CXM, CIP, GEN, NIT, TMP	+	-	+	+	+	-
16	*K. pneumoniae*	17204	urine	MDR	AMX, AMP, SAM, CTX, CXM, CIP, GEN, NIT, TZP, TMP	-	-	+	-	+	-
17	*K. pneumoniae*	17086	urine	MDR	AMX, AMP, SAM, CTX, CXM, CIP, GEN, NIT, TZP, TMP	-	-	+	-	+	-
18	*K. pneumoniae*	17171	urine	MDR	AMX, AMP, SAM, CXM, CIP, NIT, TZP, TMP	-	-	+	+	+	-

19	*K. pneumoniae*	17117	urine	MDR	AMP, CXM, CIP	+	+	+	+	+	-
20	*K. pneumoniae*	16961	urine	MDR	AMX, AMP, SAM, CTX, CXM, CIP, GEN, NIT	+	+	+	-	+	-
21	*K. pneumoniae*	17235	urine	MDR	AMX, AMP, SAM, CTX, CXM, CIP, GEN, NIT, TMP	+	-	+	+	+	-
22	*K. pneumoniae*	17281	urine	MDR	AMX, AMP, SAM, CTX, CXM, CIP, GEN, NIT, TZP, TMP	-	-	+	-	+	-
23	*K. pneumoniae*	17389	urine	MDR	AMX, AMP, SAM, CTX, CXM, CIP, TMP	-	-	+	-	+	-
24	*K. pneumoniae*	17387	urine	MDR	AMX, AMP, SAM, CTX, CXM, CIP, GEN, TMP	-	+	+	+	+	-
25	*K. pneumoniae*	17670	urine	MDR	AMX, AMP, SAM, CTX, CXM, CIP, GEN, NIT, TZP, TMP	-	-	+	-	+	-
26	*K. pneumoniae*	17578	urine	MDR	AMX, AMP, SAM, CFP-SUL, CTX, CXM, CIP, GEN, NIT, TZP, TMP	+	-	+	+	+	-
27	*K. pneumoniae*	17662	blood	MDR	AMX, AMP, SAM, CTX, CXM, CIP, GEN, PIP, TZP	-	-	+	-	+	-
28	*K. pneumoniae*	17654	urine	MDR	AMX, AMP, SAM, CFP-SUL, CTX, CXM, CIP, GEN, NIT, TZP, TMP	-	-	-	-	+	-
29	*K. pneumoniae*	17728	urine	MDR	AMX, AMP, SAM, CXM, CIP, GEN, NIT, TMP	-	-	+	+	+	-
30	*K. pneumoniae*	17528	blood	MDR	AMK, AMX, AMP, SAM, CTX, CXM, CIP, GEN, PIP, TZP	+	+	+	+	+	-
31	*K. pneumoniae*	17766	bronchus	MDR	AMX, AMP, SAM, CTX, CXM, PIP, TZP	+	-	+	-	+	-
32	*K. pneumoniae*	17698	bronchus	MDR	AMK, AMX, AMP, SAM, CTX, CXM, CIP, PIP	-	-	+	-	+	-
33	*K. pneumoniae*	17972	bronchus	-	AMP, CIP	+	+	-	-	-	+
34	*K. pneumoniae*	17768	bronchus	MDR	AMX, AMP, SAM, CFP-SUL, CTX, CXM, CIP, GEN, PIP, TZP	-	-	+	-	+	-
35	*K. pneumoniae*	17864	wound	MDR	AMX, AMP, SAM, CTX, CXM, CIP, GEN, PIP	-	-	+	-	+	-
36	*K. pneumoniae*	17871	urine	MDR	AMX, AMP, SAM, CTX, CXM, CIP, TMP	-	-	-	-	+	-
37	*K. pneumoniae*	17968	bronchus	-	AMP	+	+	+	-	+	-
38	*K. pneumoniae*	17888	urine	MDR	AMP, CIP, TMP	-	-	+	-	+	-
39	*K. pneumoniae*	17903	pus	MDR	AMX, AMP, SAM, CTX, CXM, CIP, GEN, PIP, TZP	-	-	+	-	+	-
40	*K. pneumoniae*	18018	urine	MDR	AMX, AMP, SAM, CTX, CXM, CIP, GEN, NIT, TZP, TMP	-	-	-	-	-	-
41	*K. pneumoniae*	18046	urine	MDR	AMX, AMP, SAM, CFP-SUL, CTX, CXM, CIP, GEN, NIT, TZP, TMP	-	-	+	-	+	-
41	*K. pneumoniae*	18506	urine	-	AMP	-	-	+	-	+	-
43	*K. pneumoniae*	18202	bronchus	MDR	AMX, AMP, SAM, CTX, CXM, CIP, TZP	-	-	+	-	+	-
44	*K. pneumoniae*	18241	urine	MDR	AMX, AMP, SAM, CTX, CXM, CIP, GEN, NIT, TZP, TMP	-	-	+	-	+	-
45	*K. pneumoniae*	18555	urine	MDR	AMX, AMP, SAM, CTX, CXM, CIP, GEN, NIT, TZP, TMP	-	-	+	-	+	-
46	*K. pneumoniae*	18181	urine	MDR	AMX, AMP, SAM, CTX, CXM, CIP, GEN, NIT, TZP, TMP	-	-	-	-	+	-
47	*K. pneumoniae*	18326	bronchus	MDR	AMX, AMP, SAM, CXM, CIP, TZP	+	-	+	+	+	-
48	*K. pneumoniae*	18322	bronchus	MDR	AMX, AMP, SAM, CTX, CXM, CIP, GEN	-	-	+	-	+	-
49	*K. pneumoniae*	18537	wound	MDR	AMK, AMX, AMP, SAM, CFP-SUL, CTX, CXM, CIP, GEN, TZP	-	-	+	+	+	-
50	*K. pneumoniae*	18534	pus	MDR	AMX, AMP, SAM, CFP-SUL, CTX, CXM, TZP	-	-	-	-	+	+

51	*K. pneumoniae*	18411	gall	MDR	AMX, AMP, SAM, CTX, CXM, CIP, GEN	-	-	+	-	+	-
52	*K. pneumoniae*	18412	gall	-	AMP	-	-	+	-	+	+
53	*K. pneumoniae*	18633	gall	MDR	AMX, AMP, SAM, CTX, CXM, CIP, GEN, TZP	-	-	+	+	+	-
54	*K. pneumoniae*	18608	urine	-	AMP, CIP	+	+	+	-	+	-
55	*K. pneumoniae*	18779	abdomen	MDR	AMX, AMP, SAM, CFP-SUL, CTX, CXM, CIP, TZP	-	-	+	-	+	-
56	*K. pneumoniae*	18758	urine	MDR	AMX, AMP, SAM, CTX, CXM, CIP, GEN, NIT, TZP, TMP	-	-	-	-	-	-
57	*K. pneumoniae*	18343	biopsy	MDR	AMX, AMP, SAM, CTX, CXM, CIP	-	-	+	-	+	-
58	*K. pneumoniae*	18825	phlegm	MDR	AMX, AMP, SAM, CFP-SUL, CTX, CXM, CIP, GEN, TZP	-	-	+	-	+	-
59	*K. pneumoniae*	18861	bronchus	-	AMP	+	+	+	-	+	-
60	*K. pneumoniae*	18787	urine	MDR	AMX, AMP, SAM, CTX, CXM, CIP, GEN, NIT, TZP, TMP	-	-	+	-	+	-
61	*K. pneumoniae*	18753	pus	MDR	AMX, AMP, SAM, CTX, CXM, CIP	+	+	+	+	+	-
62	*K. pneumoniae*	18812	abdomen	MDR	AMX, AMP, SAM, CTX, CXM, CIP, GEN, TZP	-	-	+	-	+	-
63	*K. pneumoniae*	18834	urine	MDR	AMX, AMP, SAM, CIP, GEN, NIT, TMP	-	-	-	-	-	-
64	*K. pneumoniae*	19058	urine	MDR	AMX, AMP, SAM, CTX, CXM, CIP, TZP, TMP	-	-	+	-	+	-
65	*K. pneumoniae*	19000	urine	-	AMP	-	-	-	-	-	-
66	*K. pneumoniae*	19108	blood	MDR	AMX, AMP, SAM, CTX, CXM, CIP, GEN, TZP	-	-	+	-	+	-
67	*K. pneumoniae*	18863	pleura	MDR	AMX, AMP, SAM, CTX, CXM, CIP, GEN	-	-	+	-	+	-
68	*K. pneumoniae*	19016	bronchus	-	AMX, AMP, SAM	+	+	+	-	+	-
69	*K. pneumoniae*	19245	urine	MDR	AMP, CXM, CIP, NIT, TMP	+	+	+	+	+	-
70	*K. pneumoniae*	19295	blood	-	AMP	+	+	-	-	+	-
71	*K. pneumoniae*	19401	bronchus	MDR	AMX, AMP, SAM, CTX, CXM, CIP, GEN	-	-	+	-	+	-
72	*K. pneumoniae*	19589	bronchus	MDR	AMX, AMP, SAM, CTX, CXM, CIP, TZP	-	-	+	-	+	-
73	*K. pneumoniae*	19314	urine	-	AMP	+	+	+	-	+	-
74	*K. pneumoniae*	19594	trachea	-	AMP	+	+	+	-	+	+
75	*K. pneumoniae*	19540	gall	-	AMP	-	-	+	-	+	-
76	*K. pneumoniae*	19425	urine	MDR	AMX, AMP, SAM, CTX, CXM, GEN, NIT, TMP	+	+	+	-	+	-
77	*K. pneumoniae*	19426	urine	MDR	AMX, AMP, SAM, CTX, CXM, CIP, GEN, TZP, TMP	+	-	+	-	+	-
78	*K. pneumoniae*	19642	urine	-	AMP	+	+	+	-	+	-
79	*K. pneumoniae*	19615	urine	MDR	AMX, AMP, SAM, CTX, CXM, CIP, GEN, NIT, TZP, TMP	-	-	+	-	+	-
80	*K. pneumoniae*	19776	urine	MDR	AMX, AMP, SAM, CTX, CXM, CIP, TZP, TMP	-	-	-	-	-	-
81	*K. pneumoniae*	19750	bronch	MDR	AMX, AMP, SAM, CTX, CXM, CIP, GEN	-	-	+	-	+	-
82	*K. pneumoniae*	19749	bronchus	-	AMP	-	-	-	-	-	-
83	*K. pneumoniae*	20089	urine	-	AMP, CIP	+	+	-	-	-	-
84	*K. pneumoniae*	20034	urine	MDR	AMX, AMP, SAM, CTX, CXM, CIP, GEN, NIT, TZP, TMP	-	-	-	-	-	-
85	*K. pneumoniae*	19834	blood	-	AMX, AMP, SAM, CIP	+	+	+	-	+	-

86	*K. pneumoniae*	20194	urine	MDR	AMX, AMP, SAM, CTX, CXM, CIP, GEN, NIT, TZP, TMP	-	-	+	-	+	-
87	*K. pneumoniae*	20190	urine	MDR	AMX, AMP, SAM, CTX, CXM, CIP, NIT, TMP	-	-	-	-	-	-
88	*K. pneumoniae*	20261	urine	MDR	AMX, AMP, SAM, CTX, CXM, CIP, GEN, NIT, TZP, TMP	-	-	+	-	+	-
89	*K. pneumoniae*	20050	bronchus	-	AMP	+	+	+	-	+	-
90	*K. oxytoca*	20172	urine	-	AMP	+	+	-	-	+	-
91	*K. oxytoca*	16658	urine	MDR	AMP, AMC, CFP, CTX, CXM, CIP, GEN, NIT, TZP, TMP	-	+	-	-	-	+
92	*K. oxytoca*	16660	urine	-	AMP	-	+	-	-	-	+
93	*K. oxytoca*	16409	bronchus	-	AMP	-	+	+	-	+	-
94	*K. oxytoca*	16807	urine	-	AMP, CXM	-	-	-	-	+	-
95	*K. oxytoca*	17293	biopsy	-	AMP	-	+	-	-	+	-
96	*K. oxytoca*	17340	urine	-	AMP, CIP	-	-	-	-	-	+
97	*K. oxytoca*	18973	urine	-	AMP	+	+	-	-	+	+
98	*K. oxytoca*	17834	pus	-	AMP	+	-	-	-	+	-
99	*K. oxytoca*	20037	urine	-	AMP	+	-	-	+	+	+
100	*K. oxytoca*	20198	bronchus	-	AMP	-	-	-	-	-	+

Supplementary Table S3. Characterization of klebicin resistant *K. quasipneumoniae* subsp. *similipneumoniae* SB30 mutants obtained by transposon mutagenesis.

Mutant No.	Selected by resistance to :	Resistant to klebicins:	Mutation	GeneBank	Complementation
#1	KpneM	KpneM2, KvarM,	FhuA	CDN05011.1	Yes
#2	KpneM	KpneM2, KvarM	FhuA	CDN05011.1	NT
#3	KpneM	KpneM2, KvarM	FhuA	CDN05011.1	NT
#4	KpneM	KpneM2, KvarM, KpneA, KaerA	TonB	CDN07115.1	Yes
#7	KpneM2	KvarM, KpneM	FhuA	CDN05011.1	NT
#14	KpneM2	KvarM, KpneM	FhuA	CDN05011.1	NT
#15	KpneM2	KvarM, KpneM	FhuA	CDN05011.1	NT
#16	KpneM2	KvarM, KpneM	FhuA	CDN05011.1	NT
#17	KpneM2	KvarM, KpneM	FhuA	CDN05011.1	NT
#18	KpneM2	KpneM, KvarM, KaerA, (partially resistant to KpneA)	ExbB	CDN08391.1	NT
#12	KvarM	KpneM, KpneM2	FhuA	CDN05011.1	NT
#13	KvarM	KpneM, KpneM2, KaerA, (partially resistant to KpneA)	ExbB	CDN08391.1	NT
#10	KpneA	KpneM2, KpneM, KvarM, KaerA	ExbB	CDN08391.1	Yes (ExbBD)
#11	KpneA	KvarIa, KaerA	OmpC	CDN07573	NT
#8	KaerA	KpneM2, (partially resistant to KvarM, KpneA, KpneM)	ExbB	CDN08391.1	NT

**#9**	KaerA	KvarIa, KpneA	OmpC	CDN07573.1	Yes
**#20**	KvarIa	KpneA, KaerA	FimB	CDN08256.1	No

Supplementary Table S4. *Klebsiella* strains from culture collections used in the study.

**Strain**	**Culture collection number**	**Strain characteristics**	**Growing temperature**
Klebsiella pneumoniae subsp. *pneumoniae*	NCTC 13368, DSM 26371, ATCC 700603	ESBL (SHV-18 control strain)	28 °C
Klebsiella pneumoniae	DSM 789, ATCC 4352		37 °C
Klebsiella pneumoniae subsp. *pneumoniae*	DSM 9377, ATCC 13887		37 °C
Klebsiella pneumoniae subsp. *rhinoscleromatis*	DSM 16231, ATCC 13884		37 °C
Klebsiella pneumoniae subsp. *ozaenae*	DSM 16358, ATCC 11296		28 °C
Klebsiella pneumoniae	NCTC 13439	VIM-1 metallo-carbapenemase	37 °C
Klebsiella pneumoniae	NCTC 13440	VIM-1 metallo-carbapenemase	37 °C
Klebsiella pneumoniae	NCTC 13443, CCUG 68728	New Delhi metallo-carbapenemase	37 °C
Klebsiella pneumoniae	NCTC 13442, CCUG 68727	OXA-48 carbapenemase	37 °C
Klebsiella pneumoniae	NCTC 13809, ATCC BAA-1705	KPC-producing strain, MHT positive	37 °C
Klebsiella pneumoniae	NCTC 13810, ATCC BAA-1706	Resistant to carbapenems by mechanisms other than carbapenemase, MHT negative	37 °C
Klebsiella pneumoniae	NCTC 13438	KPC-3 carbapenemase	37 °C
Klebsiella quasipneumoniae subsp. *quasipneumoniae*	DSM 28211		37 °C
Klebsiella quasipneumoniae subsp. *similipneumoniae*	DSM 28212		37 °C
Klebsiella oxytoca	DSM 5175, ATCC 13182		37 °C
Klebsiella oxytoca	DSM 6673, ATCC 43863		37 °C
Klebsiella variicola	DSM 15968, ATCC BAA-830		28 °C
Klebsiella aerogenes	DSM 30053		30 °C
Klebsiella aerogenes	DSM 12058		30 °C

Supplementary Table S5. Primers used for amplification of genes used in complementation assays. Restriction endonuclease sites are in italics, primer binding sequences are in bold.

Gene	Primer	Sequence	pACYC184 cloning
ExbB	ExbB Eco88I fwd	AAA*CTCGGG***TTGATGAACCTGTTTTTATACGTCT**	Eco88I-Eco81I
	ExbB Eco81I rev	AAA*CCTGAGG***TCAACCTACCCGTAATTTCTGCG**	
ExbBD	ExbB Eco88I fwd	AAA*CTCGGG***TTGATGAACCTGTTTTTATACGTCT**	Eco88I-Eco81I
	ExbD Eco81I rev	AAA*CCTGAGG***TTATTTGGCTTTGACGGTCTC**	
FhuA	FhuA Eco81I fwd	AAA*CCTCAGG***TTTAAGCCCTAAGACCAGACCC**	Eco81I
	FhuA Eco 81I rev	AAA*CCTGAGG***TTAGAAACGGAAGGTGGCGGTG**	
FimB	FimB Eco88I fwd	AAA*CTCGGG***GCTCCCGTAGCAAATAAAAACG**	Eco88I-Eco81I
	FimB Eco81I rev	AAA*CCTGAGG***TTACTGAAGCAGCGACAGGCG**	
OmpC	OmpC Eco88I fwd	AAA*CTCGGG***CTTGTGGCTGAACGACTCATCA**	Eco88I-Eco81I
	OmpC Eco81I rev	AAA*CCTGAGG***TTAGAACTGGTAAACCAGGCCC**	
TonB	TonB PsyI fwd	AAA*GACCGGGTC***GGCAAAGCTCCTTATCAATAAACA**	BseSI-PsyI
	TonB BseSI rev	AAA*GTGCCC***TCAGTTAATCTCGACGCCGTTG**	

Supplementary Text S1. Purification of Klebicins.

KpneM.

A small portion of frozen leaf tissue was homogenized with chilled mortar and pestle in liquid nitrogen. Prepared powder was mixed with cold extraction buffer (50 mM NaH_2PO_4/Na_2HPO_4, 30 mM NaCl, pH 5.0) at a ratio of 1 g of plant material to 5 ml of buffer. The crude extract kept at 20-25 °C for 10-15 min. Cell debris were removed by centrifugation at 3220 *g,* at 4 °C for 20 min. Pellets were discarded and the supernatant was filtered by passing solution through membrane filters (pore sizes 5 µm and 0.45 µm). Ammonium sulphate was added up to 0.70 M and pH of solution adjusted to 6. Formed precipitate removed by centrifugation at 3220 *g,* at 4 °C for 5 min. The supernatant taken as total soluble protein and applied for purification in two steps.

At the first purification step the chromatography column was filled with Phenyl sepharose FF resin (GE Healthcare Life Sciences, Uppsala, Sweden) and pre-equilibrated with cold buffer (50 mM NaH_2PO_4/Na_2HPO_4, 0.70 M $(NH_4)_2SO_4$, pH 6.0). Protein solution was loaded to column and the Phenyl sepharose bounded protein fraction was eluted by washing with elution buffer (50 mM NaH_2PO_4/Na_2HPO_4, 0.28 M $(NH_4)_2SO_4$, pH 6.0). Collected protein fraction replaced to the diafiltrating concentrator (10 kDa) and centrifuged at 3220 *g* until the volume of protein solution decreased 8-10 folds. Concentrate was diluted up to a primary volume with buffer containing 50 mM NaH_2PO_4/Na_2HPO_4 (pH 8.0). Procedure was repeat till conductivity decreased below 10 mS/cm and protein solution subjected to the final purification step using Q sepharose FF resin (GEHealthcare Life Sciences, Uppsala, Sweden). Chromatography media was pre-equilibrated with cold buffer (50 mM NaH_2PO_4/Na_2HPO_4, pH 8.0). Protein solution was loaded to column and Q sepharose unbounded protein was collected in flow through fraction. After KpneM was freeze-dried and applied for analysis.

KpneM2.

A small portion of frozen leaf tissue was homogenized with chilled mortar and pestle in liquid nitrogen. Prepared powder was mixed with cold extraction buffer (50 mM NaH_2PO_4/Na_2HPO_4, 30 mM NaCl, pH 5.0) at a ratio of 1 g of plant material to 5 ml of buffer. The crude extract kept at 20-25 °C for 10-15 min. Cell debris were removed by centrifugation at 3220 *g,* at 4 °C for 20 min. Pellets were discarded and the supernatant was filtered by passing solution through membrane filters (pore sizes 5 µm and 0.45 µm). Ammonium sulphate was added up to 0.70 M and pH of solution adjusted to 6. Formed precipitate removed by centrifugation at 3220 *g,* at 4 °C for 5 min. The supernatant taken as total soluble protein and applied for purification in two steps.

At the first purification step the chromatography column was filled with Phenyl sepharose FF resin (GE Healthcare Life Sciences, Uppsala, Sweden) and pre-equilibrated with cold buffer (50 mM NaH_2PO_4/Na_2HPO_4, 0.70 M $(NH_4)_2SO_4$, pH 6.0). Protein solution was loaded to column and the Phenyl sepharose bounded protein fraction was eluted by washing with elution buffer (50 mM NaH_2PO_4/Na_2HPO_4, 0.42 M $(NH_4)_2SO_4$, pH 6.0). Collected protein fraction replaced to the diafiltrating concentrator (10 kDa) and centrifuged at 3220 *g* until the volume of protein solution decreased 8-10 folds. Concentrate was diluted up to a primary volume with buffer containing 50 mM NaH_2PO_4/Na_2HPO_4 (pH 8.0). Procedure was repeat till conductivity decreased below 10 mS/cm and protein solution subjected to the final purification step using Q sepharose FF resin (GEHealthcare Life Sciences, Uppsala, Sweden). Chromatography media was pre-equilibrated with cold buffer (50 mM NaH_2PO_4/Na_2HPO_4, pH 8.0). Protein solution was loaded to column and Q sepharose unbounded protein was collected in flow through fraction. After KpneM2 was freeze-dried and applied for analysis.

KvarM.

A small portion of frozen leaf tissue was homogenized with chilled mortar and pestle in liquid nitrogen. Prepared powder was mixed with cold extraction buffer (50 mM NaH_2PO_4/Na_2HPO_4, pH 5.0) at a ratio of 1 g of plant material to 5 ml of buffer. The crude extract kept at 20-25 °C for 10-15 min. Cell debris were removed by centrifugation at 3220 *g,* at 4 °C for 20 min. Pellets were discarded and the supernatant was filtered by passing solution through membrane filters (pore sizes 5 µm and 0.45 µm). Ammonium sulphate was added up to 0.95 M and pH of solution adjusted to 6.

Formed precipitate removed by centrifugation at 3220 *g,* at 4 °C for 5 min. The supernatant taken as total soluble protein and applied for purification in two steps.

At the first purification step the chromatography column was filled with Phenyl sepharose FF resin (GE Healthcare Life Sciences, Uppsala, Sweden) and pre-equilibrated with cold buffer (50 mM NaH_2PO_4/Na_2HPO_4, 0.95 M $(NH_4)_2SO_4$, pH 6.0). Protein solution was loaded to column and the Phenyl sepharose bounded protein fraction was eluted by washing with elution buffer (50 mM NaH_2PO_4/Na_2HPO_4, 0.62 M $(NH_4)_2SO_4$, pH 6.0). Collected protein fraction replaced to the diafiltrating concentrator (10 kDa) and centrifuged at 3220 *g* until the volume of protein solution decreased 8-10 folds. Concentrate was diluted up to a primary volume with buffer containing 50 mM NaH_2PO_4/Na_2HPO_4 (pH 8.0). Procedure was repeat till conductivity decreased below 10 mS/cm and protein solution subjected to the final purification step using Q sepharose FF resin (GEHealthcare Life Sciences, Uppsala, Sweden). Chromatography media was pre-equilibrated with cold buffer (50 mM NaH_2PO_4/Na_2HPO_4, pH 8.0). Protein solution was loaded to column and Q sepharose unbounded protein was collected in flow through fraction. After KvarM was freeze-dried and applied for analysis.

KpneA.

A small portion of frozen leaf tissue was homogenized with chilled mortar and pestle in liquid nitrogen. Prepared powder was mixed with cold extraction buffer (20 mM NaH_2PO_4/Na_2HPO_4, 30 mM NaCl, pH 5.0) at a ratio of 1 g of plant material to 5 ml of buffer. The crude extract kept at 20-25 °C for 10-15 min. Cell debris were removed by centrifugation at 3220 *g,* at 4 °C for 20 min. Pellets were discarded and the supernatant was filtered by passing solution through membrane filters (pore sizes 5 µm and 0.45 µm). Ammonium sulphate was added up to 1.50 M and pH of solution adjusted to 6. Formed precipitate removed by centrifugation at 3220 *g,* at 4 °C for 5 min. The supernatant taken as total soluble protein and applied for purification in two steps.

At the first purification step the chromatography column was filled with Phenyl sepharose FF resin (GE Healthcare Life Sciences, Uppsala, Sweden) and pre-equilibrated with cold buffer (50 mM NaH_2PO_4/Na_2HPO_4, 1.50 M $(NH_4)_2SO_4$, pH 6.0). Protein solution was loaded to column and the Phenyl

sepharose bounded protein fraction was eluted by washing with elution buffer (50 mM NaH_2PO_4/Na_2HPO_4, 0.90 M $(NH_4)_2SO_4$, pH 6.0). Collected protein fraction replaced to the diafiltrating concentrator (10 kDa) and centrifuged at 3220 *g* until the volume of protein solution decreased 8-10 folds. Concentrate was diluted up to a primary volume with buffer containing 20 mM NaH_2PO_4/Na_2HPO_4, 20 mM Sodium citrate (pH 4.5). Procedure was repeat till conductivity decreased below 9 mS/cm and protein solution subjected to the final purification step using SP sepharose FF resin (GEHealthcare Life Sciences, Uppsala, Sweden). Chromatography media was pre-equilibrated with cold buffer (20 mM NaH_2PO_4/Na_2HPO_4, 20 mM Citric acid, pH 4.5). Protein solution was loaded to column and SP sepharose bounded protein fraction was eluted by linear gradient of cold washing buffer additionally containing 500 mM of NaCl. After KpneA was freeze-dried and applied for analysis.

KaerA.

A small portion of frozen leaf tissue was homogenized with chilled mortar and pestle in liquid nitrogen. Prepared powder was mixed with cold extraction buffer (20 mM NaH_2PO_4/Na_2HPO_4, 20 mM Citric acid, pH 5.0) at a ratio of 1 g of plant material to 5 ml of buffer. The crude extract kept at 20-25 °C for 10-15 min. Cell debris were removed by centrifugation at 3220 *g,* at 4 °C for 20 min. Pellets were discarded and the supernatant was filtered by passing solution through membrane filters (pore sizes 5 µm and 0.45 µm). The pH of solution adjusted to 4.5 and formed precipitate removed by centrifugation at 3220 *g,* at 4 °C for 5 min. The supernatant taken as total soluble protein and applied for purification in two steps.

At the first purification step the chromatography column was filled with SP sepharose FF resin (GE Healthcare Life Sciences, Uppsala, Sweden) and pre-equilibrated with cold buffer (20 mM NaH_2PO_4/Na_2HPO_4, 20 mM Citric acid, pH 4.5). Protein solution was loaded to column and SP sepharose bounded protein fraction was eluted by linear gradient of cold washing buffer additionally containing 500 mM of NaCl. Collected protein fraction replaced to the diafiltrating concentrator (10 kDa) and centrifuged at 3220 *g* until the volume of protein solution decreased 8-10 folds. Concentrate was diluted up to a primary volume with buffer containing 20 mM NaH_2PO_4/Na_2HPO_4, (pH 8.0). Procedure was repeat till conductivity decreased below 8 mS/cm and protein solution subjected to the final purification step using Q sepharose FF resin (GEHealthcare Life Sciences, Uppsala, Sweden).

Chromatography media was pre-equilibrated with cold buffer (20 mM NaH_2PO_4/Na_2HPO_4, pH 8.0). Protein solution was loaded to column and Q sepharose unbounded protein was collected in flow through fraction. After KaerA was freeze-dried and applied for analysis.

KvarIa.

A small portion of frozen leaf tissue was homogenized with chilled mortar and pestle in liquid nitrogen. Prepared powder was mixed with cold extraction buffer (20 mM NaH_2PO_4/Na_2HPO_4, 30 mM NaCl, pH 5.0) at a ratio of 1 g of plant material to 5 ml of buffer. The crude extract kept at 20-25 °C for 10-15 min. Cell debris were removed by centrifugation at 3220 *g,* at 4 °C for 20 min. Pellets were discarded and the supernatant was filtered by passing solution through membrane filters (pore sizes 5 µm and 0.45 µm). Ammonium sulphate was added up to 1.35 M and pH of solution adjusted to 6. Formed precipitate removed by centrifugation at 3220 *g,* at 4 °C for 5 min. The supernatant taken as total soluble protein and applied for purification in two steps.

At the first purification step the chromatography column was filled with Phenyl sepharose FF resin (GE Healthcare Life Sciences, Uppsala, Sweden) and pre-equilibrated with cold buffer (50 mM NaH_2PO_4/Na_2HPO_4, 1.35 M $(NH_4)_2SO_4$, pH 6.0). Protein solution was loaded to column and the Phenyl sepharose bounded protein fraction was eluted by washing with elution buffer (50 mM NaH_2PO_4/Na_2HPO_4, 0.81 M $(NH_4)_2SO_4$, pH 6.0). Collected protein fraction replaced to the diafiltrating concentrator (10 kDa) and centrifuged at 3220 *g* until the volume of protein solution decreased 8-10 folds. Concentrate was diluted up to a primary volume with buffer containing 20 mM NaH_2PO_4/Na_2HPO_4, 20 mM Sodium citrate (pH 4.5). Procedure was repeat till conductivity decreased below 8 mS/cm and protein solution subjected to the final purification step using SP sepharose FF resin (GEHealthcare Life Sciences, Uppsala, Sweden). Chromatography media was pre-equilibrated with cold buffer (20 mM NaH_2PO_4/Na_2HPO_4, 20 mM Citric acid, pH 4.5). Protein solution was loaded to column and SP sepharose bounded protein fraction was eluted by linear gradient of cold washing buffer additionally containing 500 mM of NaCl. After KvarIa was freeze-dried and applied for analysis.

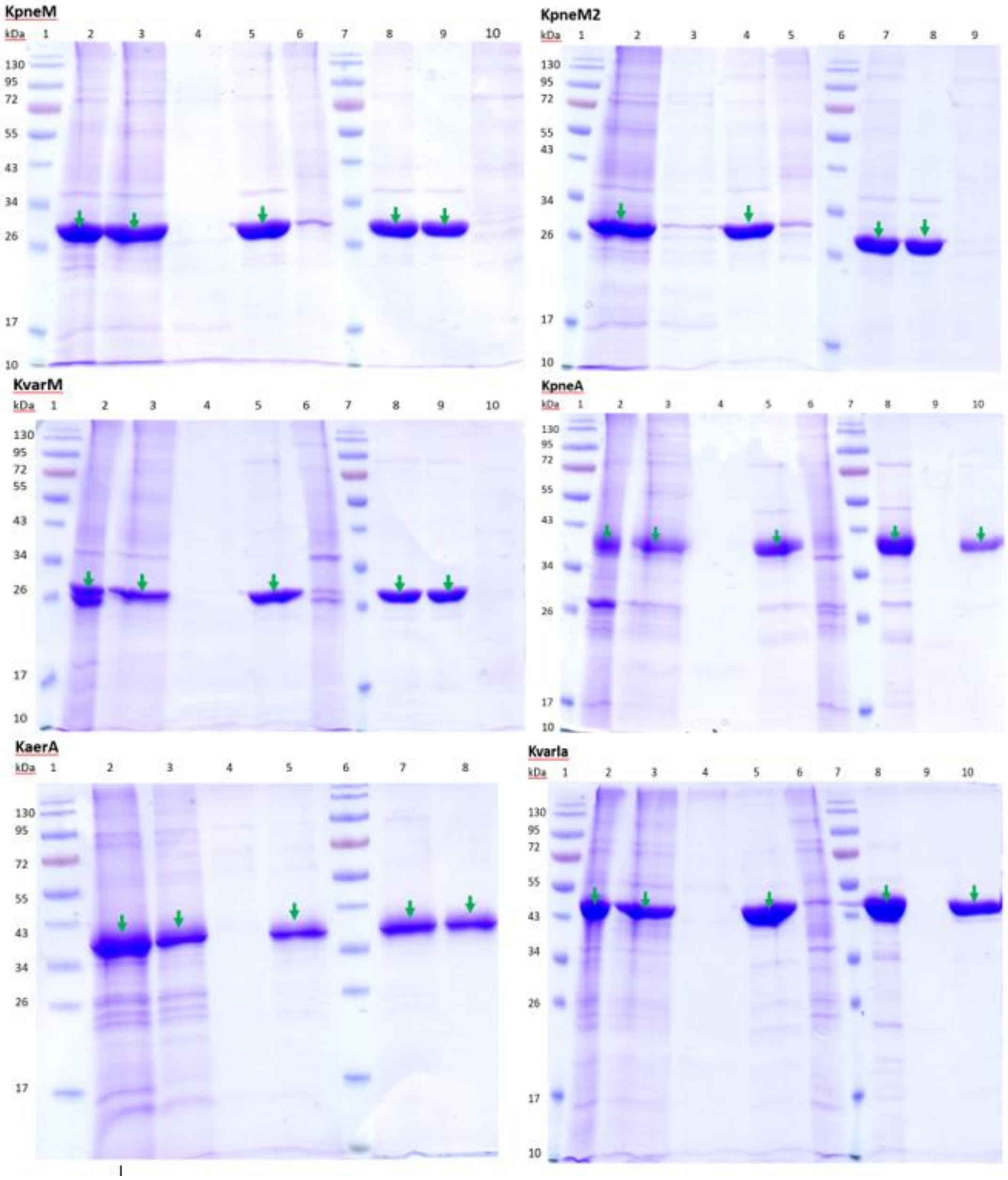

Supplementary Figure S1. Coomassie stained SDS-PAGE gels illustrating the purification flow of plant – produced klebicins. KpneM – lane 1 and 7 - PageRuler™ Prestained protein ladder, lane 2 – crude extract, lane 3 – total soluble proteins loaded on Phenyl sepharose, lane 4 – flow through Phenyl sepharose, lane 5 – KpneM eluate (after Phenyl sepharose), lane 6 – impurities eluate (after Phenyl sepharose), lane 8 – proteins loaded on Q sepharose, lane 9 - KpneM flow through Q sepharose, lane 10 - impurities eluate (after Q sepharose). **KpneM2** – lane 1 and 6 - PageRuler™ Prestained protein ladder, lane 2 – total soluble proteins loaded on Phenyl sepharose, lane 3 – flow through Phenyl sepharose, lane 4 – KpneM2 eluate (after Phenyl sepharose), lane 5 – impurities eluate (after Phenyl sepharose), lane 7 – proteins loaded on Q sepharose, lane 8 – KpneM2 flow through Q sepharose, lane 9 - impurities eluate (after Q sepharose). **KvarM** – lane 1 and 7 - PageRuler™ Prestained protein ladder, lane 2 – crude extract, lane 3 - total soluble proteins loaded on Phenyl sepharose, lane 4 – flow through Phenyl sepharose, lane 5 – KvarM eluate (after Phenyl sepharose), lane 6 – impurities eluate (after Phenyl sepharose), lane 8 – proteins loaded on Q sepharose, lane 9 – KvarM flow through Q sepharose, lane 10 - impurities eluate (after Q sepharose). **KpneA** – lane 1 and 7 - PageRuler™ Prestained protein ladder, lane 2 – crude extract, lane 3 – total soluble proteins loaded on Phenyl sepharose, lane 4 – flow through Phenyl sepharose, lane 5 – KpneA eluate (after Phenyl sepharose), lane 6 – impurities eluate (after Phenyl sepharose), lane 8 – proteins loaded on SP sepharose, lane 9 – flow through SP sepharose, lane 10 - KpneA eluate (after SP sepharose). **KaerA** – lane 1 and 6 - PageRuler™ Prestained protein ladder, lane 2 – crude extract, lane 3 – total soluble proteins loaded on SP sepharose, lane 4 – flow through SP sepharose, lane 5 – KaerA eluate (after SP sepharose), lane 7 – proteins loaded on Q sepharose, lane 8 – KaerA flow through Q sepharose. **KvarIa** – lane 1 and 7 - PageRuler™ Prestained protein ladder, lane 2 – crude extract, lane 3 – total soluble proteins loaded on Phenyl sepharose, lane 4 – flow through Phenyl sepharose, lane 5 – KvarIa eluate (after Phenyl sepharose), lane 6 – impurities eluate (after Phenyl sepharose), lane 8 – proteins loaded on SP sepharose, lane 9 – flow through SP sepharose, lane 10 - KvarIa eluate (after SP sepharose). Green arrows mark recombinant proteins.

Supplementary figure S2. Purified *N. benthamiana*-expressed klebicins. 0.5 µg of each purified protein was resolved in 12% polyacrylamide gel for Coomassie staining.

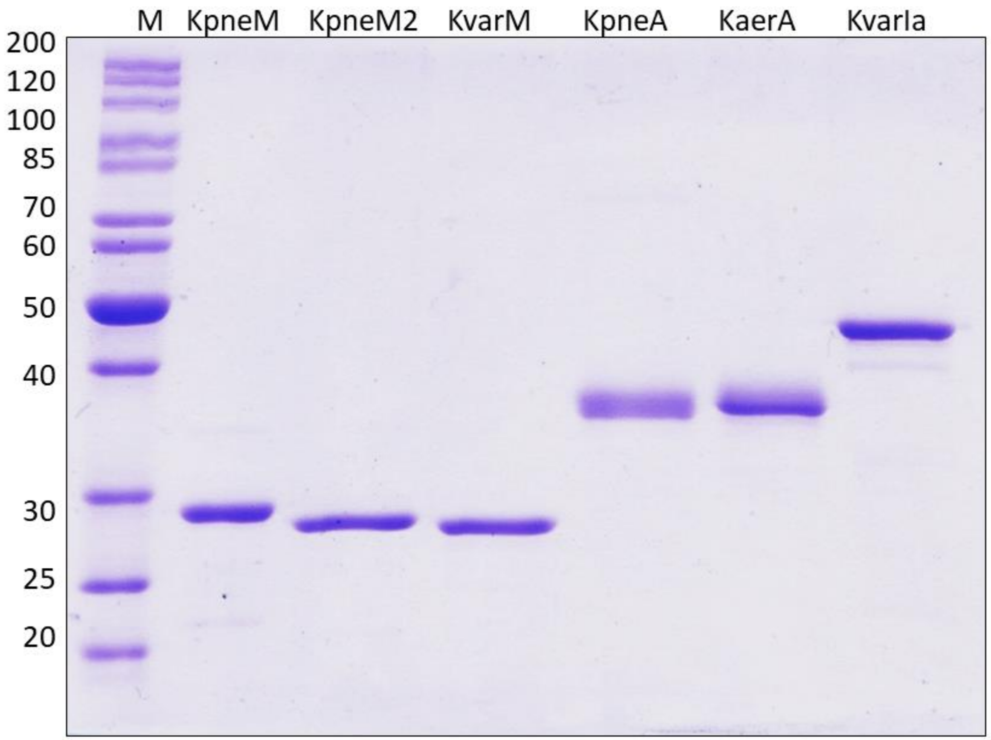

Supplementary figure S3. Impact of KvarIa treatment on the survival of *Galleria mellonella* larvae after challenge with *K. quasipneumoniae* DSM 28212. *G. melonella* larvae were infected with 12000-32000 CFU of *K. pneumoniae* DSM 28212 and treated with 10 µg of KvarIa 2 hours after infection. Larvae were incubated in Petri dishes at 37 °C up to 68 h. 20 larvae were used in each treatment group.

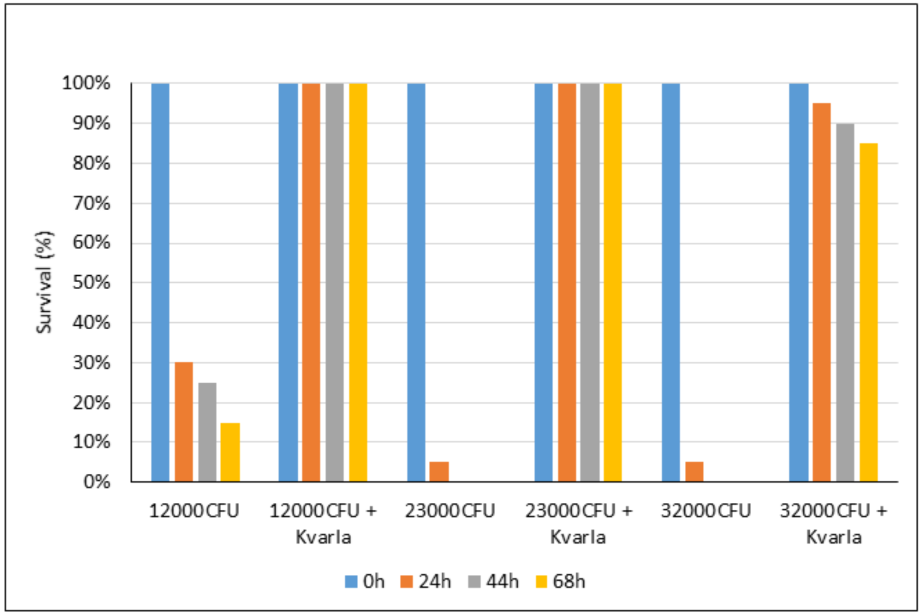

Supplementary figure S4. Clustal W amino acid sequence alignment of KvarIa, KpneIa, KoxyY and some representatives of group A pore-forming colicins. KoxyY (WP_024273778), KvarIa (KDL88409), KpneIa (BAS34675), Col28B (CAA44310.1), ColA (P04480.1), ColR (AGV40809.1), ColU (CAA72509.1), ColY (AAF82683.1).

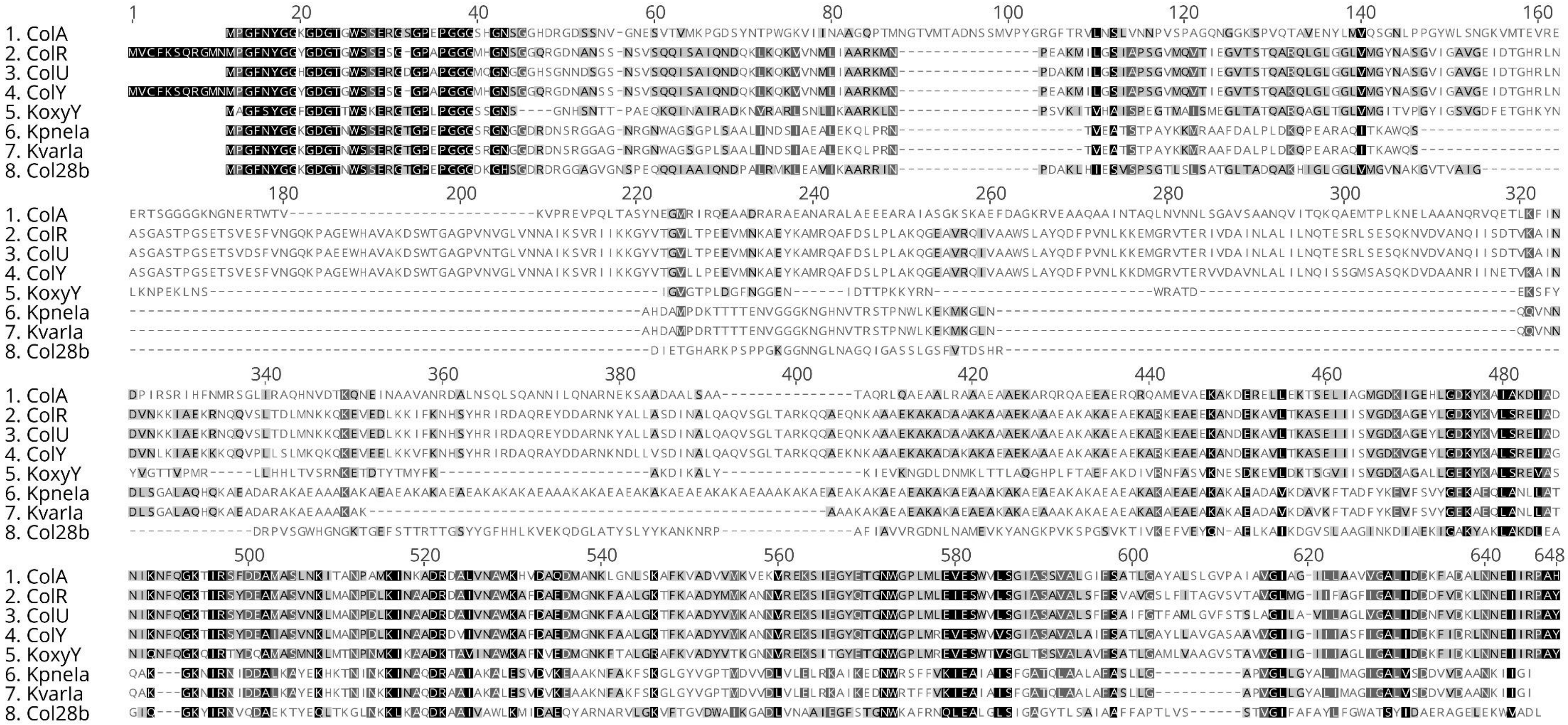

References:

1 Wiegand, I., Hilpert, K. & Hancock, R. E. Agar and broth dilution methods to determine the minimal inhibitory concentration (MIC) of antimicrobial substances. *Nat Protoc* **3**, 163-175, doi:10.1038/nprot.2007.521 (2008).
